# Directed evolution and selection of biostable l-DNA aptamers with a mirror-image DNA polymerase

**DOI:** 10.1038/s41587-022-01337-8

**Published:** 2022-06-06

**Authors:** Ji Chen, Mengyin Chen, Ting F. Zhu

**Affiliations:** 1 School of Life Sciences, Tsinghua-Peking Center for Life Sciences, Beijing Frontier Research Center for Biological Structure, Beijing Advanced Innovation Center for Structural Biology, Tsinghua University, Beijing, China; 2 School of Life Sciences, Westlake University, Hangzhou, Zhejiang China; 3 Westlake Laboratory of Life Sciences and Biomedicine, Hangzhou, Zhejiang China

**Keywords:** Nucleic-acid therapeutics, Chemical biology

## Abstract

Mirror-image aptamers made from chirally inverted nucleic acids are nuclease-resistant and exceptionally biostable, opening up opportunities for unique applications. However, the directed evolution and selection of mirror-image aptamers directly from large randomized l-DNA libraries has,﻿ to our knowledge, not been demonstrated previously. Here, we developed a ‘mirror-image selection’ scheme for the directed evolution and selection of biostable l-DNA aptamers with a mirror-image DNA polymerase. We performed iterative rounds of enrichment and mirror-image polymerase chain reaction (PCR) amplification of l-DNA sequences that bind native human thrombin, in conjunction with denaturing gradient gel electrophoresis (DGGE) to isolate individual aptamers and l-DNA sequencing-by-synthesis to determine their sequences. Based on the selected l-DNA aptamers, we designed biostable thrombin sensors and inhibitors, which remained functional in physiologically relevant nuclease-rich environments, even in the presence of human serum that rapidly degraded d-DNA aptamers. Mirror-image selection of biostable l-DNA aptamers directly from large randomized l-DNA libraries greatly expands the range of biomolecules that can be targeted, broadening their applications as biostable sensors, therapeutics and basic research tools.

## Abstract (web summary)

Mirror-image PCR and l-DNA sequencing-by-synthesis enable the directed evolution and selection of functional l-DNA aptamers.

## Main

Natural aptamers selected through systematic evolution of ligands by exponential enrichment (SELEX) or in vitro selection^1,2^ are vulnerable to degradation by nucleases ubiquitous in vitro and in vivo, limiting their practical applications as diagnostic and therapeutic tools^3^. The chirally inverted mirror-image (l-DNA or l-RNA) aptamers are nuclease-resistant and exceptionally biostable^4–6^. Their production can be readily implemented by automated oligo synthesizers with commercially available l-deoxynucleoside or l-ribonucleoside phosphoramidites^7^, making them well-suited for practical applications in diagnostics and therapeutics. Since the appreciation of their biochemical advantages over two decades ago, mirror-image aptamers have been selected primarily through an indirect scheme known as ‘selection-reflection’: the mirror-image version of the target molecule is first chemically synthesized for the selection of a natural aptamer, after which a mirror-image aptamer with the same sequence is synthesized to bind the corresponding natural target^4–6^. However, the first step of chemically synthesizing the mirror-image target molecule is often problematic, especially for proteins with large sizes, extensive posttranslational modifications (PTMs) and low in vitro folding efficiencies. In practice, most biologically important target molecules such as large proteins cannot be chemically synthesized based on current technologies^7^. As a result, only a small number of mirror-image aptamers have been discovered by selection-reflection thus far, largely restricted to targeting small molecules^4,5^, short peptides^6,8^, short RNAs^9,10^ and small proteins^11,12^, with the largest being a 110-amino acid (aa) ribonuclease from *Bacillus amyloliquefaciens* (barnase) at 12 kDa (ref. ^11^). Selections of mirror-image aptamers targeting most biologically important and yet unsynthesizable target molecules have not been realized.

**Fig. 1:**
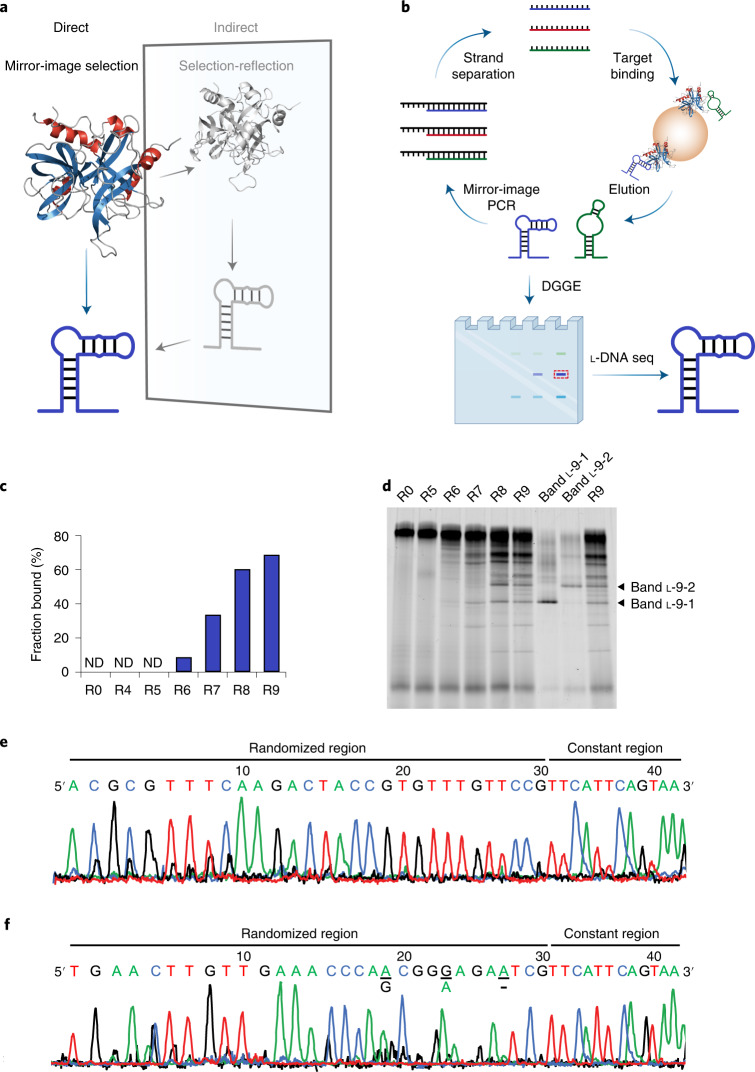
Mirror-image selection of l-DNA aptamers targeting native human thrombin. **a**, Schematic overview of the direct, mirror-image selection scheme (color), which bypasses the need for chemically synthesizing mirror-image target molecules as in the indirect, selection-reflection scheme (gray). Protein Data Bank source 1PPB (native human thrombin). **b**, Schematic overview of the procedures in the mirror-image selection scheme: selection begins with a large randomized l-DNA library (for example, of roughly 1 × 10^14^ distinct l-DNA sequences in this work) to bind immobilized protein targets such as native human thrombin; the bound l-DNA is eluted and amplified by mirror-image polymerase chain reaction (PCR); the amplified l-DNA pool is separated into single-stranded l-DNAs for the next round; after the final round of mirror-image selection, the enriched l-DNA pool is analyzed by DGGE, isolated and sequenced by l-DNA sequencing-by-synthesis. **c**, Monitoring the progress of mirror-image selection by EMSA, with the corresponding native PAGE shown in Extended Data Fig. 1b and the fraction bound determined by the ImageJ software using the band intensity of the bound l-DNA pool relative to the total lane fluorescence intensity. ND, (binding) not detected. The experiment was performed once. **d**, DGGE analysis of the corresponding l-DNA library and pools, as well as the isolated bands l-9-1 and l-9-2, re-amplified by mirror-image PCR using d-Dpo4-5m with l-dNTPs and l-DNA primers, analyzed by 10% denaturing PAGE in 2.1–4.2 M urea and 12–24% formamide, stained by SYBR Green II and scanned by the Amersham Typhoon Biomolecular Imager under the Cy2 mode. The experiment was performed twice with similar results. **e**,**f**, Sequencing chromatograms of bands l-9-1 (**e**) and l-9-2 (**f**) amplified by mirror-image PCR using d-Dpo4-5m with l-dNTPαSs and 5′-FAM-labeled l-DNA forward sequencing primer after natural CIP treatment (with the corresponding sequencing gels shown in Supplementary Fig. 7b,c, respectively). The three ambiguous nucleotide positions in the randomized region of the sequenced l-9-2 aptamer are underlined with the most probable alternative nucleotides (A and G) or deletion (−) indicated. The experiments were performed twice with similar results.

We reasoned that developing a ‘mirror-image selection’ scheme to select l-DNA aptamers directly from large randomized l-DNA libraries would bypass most of the aforementioned problems and become a far more generalizable method than the traditional selection-reflection scheme (Fig. 1a). Even with the development of new technologies that could enable the synthesis of larger and larger mirror-image target molecules^13,14^ for selection-reflection, synthesizing various complex biological targets is far more difficult (if possible at all) than preparing the molecular tools needed for mirror-image selection because the targets can become almost infinitely complex, whereas the mirror-image molecular tools need not to. Here, we set out to develop a mirror-image selection scheme for the selection of l-DNA aptamers directly from large randomized l-DNA libraries (Fig. 1b). Taking advantage of a series of our recently developed mirror-image molecular tools, we successfully selected and identified several l-DNA aptamers targeting native human thrombin.

## Results

### Designing a mirror-image selection scheme

One of the key techniques required for realizing mirror-image selection is the amplification of large randomized l-DNA libraries (Fig. 1b). We previously reported a mirror-image PCR system based on d-Dpo4-5m (a designed mutant of *Sulfolobus solfataricus* P2 DNA polymerase IV (Dpo4) to facilitate its chemical synthesis)^15,16^. Despite the high error rate and low amplification efficiency especially for long l-DNA sequences, its relatively small size has made the total chemical synthesis of large quantities of d-Dpo4-5m more practical than larger d-polymerases such as the mirror-image *Pyrococcus furiosus* (*Pfu*) DNA polymerase^14^ and thus potentially suitable for amplifying large pools of short l-DNA sequences.

Another key technique required for realizing mirror-image selection is the sequencing of enriched l-DNA aptamers. One potential approach would be to amplify l-DNAs from single molecules (similar to cell-free cloning through limiting dilution^17^) to isolate single l-DNA aptamers for downstream l-DNA sequencing^14,18^. However, the amplification efficiencies of the current mirror-image PCR systems based on mirror-image Dpo4 and *Pfu* DNA polymerase are not sufficient for amplifying single-molecule templates^14–16^. Another potential approach would be to apply high-throughput l-DNA sequencing to sequence the enriched l-DNA aptamers directly. Nevertheless, ﻿high-throughput l-DNA sequencing has not been developed with the limited mirror-image molecular tools currently available. Here, we repurposed denaturing gradient gel electrophoresis (DGGE), a technique traditionally used for analyzing microbial community compositions by separating different DNA sequences of similar lengths based on their different melting temperatures (mainly for their GC contents)^19^, to isolate the enriched l-DNA aptamers for downstream l-DNA sequencing (Fig. 1b).

### Validating the selection scheme in the natural system

**Extended Data Fig. 1:**
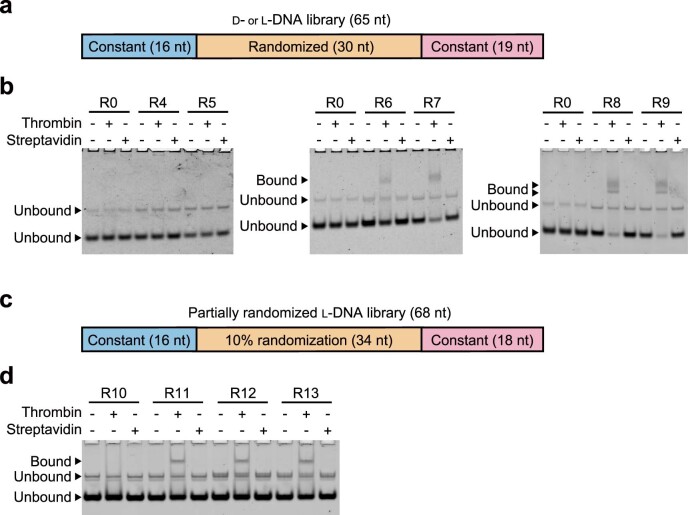
Mirror-image selection and secondary selection of l-DNA aptamers targeting native human thrombin. **a**, Design of the d- or l-DNA library for selection in this work. **b**, Monitoring the progress of mirror-image selection by EMSA with 200 nM corresponding l-DNA library and pools binding with 1 μM native human thrombin or 1 μM streptavidin, analyzed by 8% native PAGE, stained by SYBR Green II, and scanned by the Amersham Typhoon Biomolecular Imager under the Cy2 mode, with the corresponding gel quantitation results shown in Fig. 1c. The experiment was performed once. **c**, Design of the partially randomized l-DNA library for the secondary selection in this work. **d**, Monitoring the progress of secondary selection by EMSA with 200 nM corresponding partially randomized l-DNA library and pools binding with 1 μM native human thrombin or 1 μM streptavidin, analyzed by 8% native PAGE, stained by SYBR Green II, and scanned by the Amersham Typhoon Biomolecular Imager under the Cy2 mode, with the corresponding gel quantitation results shown in Fig. 3b. The experiment was performed twice with similar results.

Although Dpo4-5m has been shown to amplify short DNA sequences efficiently^15,16^, it has not been tested in the amplification of large randomized DNA libraries. Here, we prepared a large randomized d-DNA library of roughly 1 × 10^14^ distinct sequences by solid-phase oligo synthesis, with 30 randomized nucleotides flanked by two constant regions for primer binding (Extended Data Fig. 1a). We validated the ability of l-Dpo4-5m to amplify the large randomized d-DNA library (Supplementary Fig. 1a), and performed iterative rounds of selection of d-DNA aptamers targeting commercially available native human thrombin purified from plasma, against which high-affinity d-DNA aptamers have been previously selected^20,21^. The progress of selection was monitored by the electrophoretic mobility shift assay (EMSA)^22^ (Supplementary Fig. 2a), which helps to access the overall binding fraction of the sequence pool during each selection round. After six rounds of selection, we observed that roughly 70% of the d-DNA pool bound 1 μM native human thrombin, but not 1 μM streptavidin (Supplementary Fig. 2a,b). Next, we sequenced the round 6 (R6) d-DNA pools by high-throughput sequencing, which revealed the enrichment of multiple sequences, although the most abundant sequence only accounted for roughly 1.1% of the total reads (Supplementary Fig. 2c,d).

We then asked if d-DNA sequences of similar lengths could be separated by DGGE based on their different melting temperatures. We applied DGGE to analyze the R4–R6 d-DNA pools, along with the unselected R0 d-DNA library (Supplementary Fig. 3a,b). We found that while no clear band was observed in R0 and R4, single bands began to emerge in R5, with both the number and fluorescence intensity of the bands increased in R6 (Supplementary Fig. 3b). Next, we isolated a single band (d-6) from R6, accounting for roughly 1.7% of the total lane fluorescence intensity of R6 (Supplementary Fig. 3b). We amplified band d-6 by natural PCR using l-Dpo4-5m with d-deoxynucleoside triphosphates (d-dNTPs) and d-DNA primers, and analyzed the natural PCR product by another DGGE, which revealed a predominant band accounting for roughly 35% of the total lane fluorescence intensity (Supplementary Fig. 3b). We recovered the band from DGGE and analyzed its composition by high-throughput sequencing, which revealed a single sequence accounting for roughly 45% of the total reads (249,272 in 554,081 reads) (Supplementary Fig. 3c,d). In fact, the same sequence (d-6) was also found in the R6 d-DNA pool before DGGE separation, but only accounting for roughly 0.8% (ranked fourth) of the high-throughput sequencing reads (Supplementary Fig. 2c,d). Therefore, although the d-6 sequence was rather rare in the R6 d-DNA pool (roughly 1.7% revealed by DGGE, and roughly 0.8% by high-throughput sequencing, respectively), it became predominant after DGGE separation and PCR amplification by l-Dpo4-5m (roughly 35% revealed by DGGE, and roughly 45% by high-throughput sequencing, respectively).

Next, we sequenced band d-6 using the phosphorothioate approach with d-deoxynucleoside α-thiotriphosphates (d-dNTPαSs) and cleavage by 2-iodoethanol^23^, which we have adopted recently for l-DNA sequencing-by-synthesis^14^. We observed that the sequencing results were rather ambiguous due to band doubling (Supplementary Fig. 4a), a phenomenon that was primarily attributed to the presence of 3′-hydroxyl and 3′-monophosphate groups among the cleaved DNA fragments^24^. To address this issue, we treated the 2-iodoethanol-cleaved d-DNA fragments with natural calf intestinal alkaline phosphatase (CIP). We observed that most of the band doubling disappeared after natural CIP treatment, likely due to the removal of 3′-monophosphates from the cleaved d-DNA fragments, and hence the sequence of band d-6 was readily determined (Supplementary Fig. 4b). Prediction of secondary structure of the d-6 aptamer by Mfold^25^ reveals that it matches the consensus sequence of previously identified d-DNA aptamers targeting native human thrombin^20^ (Supplementary Fig. 5a). Finally, we prepared the d-6 aptamer by solid-phase oligo synthesis, and applied isothermal titration calorimetry (ITC) to measure its binding affinity with native human thrombin (without detectable autolysis that may affect the ITC results, as shown in Supplementary Fig. 6), with dissociation constant (*K*_d_) measured at 27 nM in physiological buffer (20 mM HEPES-NaOH, 150 mM NaCl, 5 mM KCl, 2 mM MgCl_2_, 1 mM CaCl_2_, pH 7.4) (Supplementary Fig. 5b). Furthermore, the d-6 aptamer formed stable complexes with native human thrombin as revealed by EMSA, and was, as expected, digestible by natural DNase I (Supplementary Fig. 5c).

### Mirror-image selection of l-DNA aptamers targeting native human thrombin

Having validated the selection scheme in the natural system, we selected l-DNA aptamers targeting native human thrombin, a biologically important target molecule for treating thrombosis^26^ and yet difficult to be chemically synthesized for its relative large size (295 aa) and glycosylation^27^. We prepared a large randomized l-DNA library of roughly 1 × 10^14^ distinct sequences by solid-phase oligo synthesis, with 30 randomized nucleotides flanked by two constant regions for primer binding, as with the d-DNA library (Extended Data Fig. 1a). We amplified the l-DNA library by mirror-image PCR using d-Dpo4-5m with l-dNTPs and l-DNA primers (Supplementary Fig. 1b), and monitored the progress of mirror-image selection by EMSA (Extended Data Fig. 1b). After nine rounds of selection, we observed that roughly 70% of the l-DNA pool bound 1 μM native human thrombin, but not 1 μM streptavidin (Fig. 1c and Extended Data Fig. 1b). We applied DGGE to analyze the R5-R9 l-DNA pools, along with the unselected R0 l-DNA library (Fig. 1d). We found that while no clear band was observed in R0 and R5, single bands began to emerge in R6, with both the number and fluorescence intensity of the bands increased from R7 to R9 (Fig. 1d). Next, we isolated two bands (l-9-1 and l-9-2) from R9, accounting for roughly 1.7% and 1.6% of the total lane fluorescence intensity of R9, respectively (Fig. 1d). We amplified bands l-9-1 and l-9-2 by mirror-image PCR using d-Dpo4-5m with l-dNTPs and l-DNA primers in two separate reactions, and analyzed the mirror-image PCR products by another DGGE, both revealing a predominant band accounting for roughly 18% and 12% of the total lane fluorescence intensity, respectively (Fig. 1d).

To sequence the enriched l-DNA aptamers, we isolated band l-9-1 for l-DNA sequencing-by-synthesis using the phosphorothioate approach with l-dNTPαSs and cleavage by 2-iodoethanol^14^. We observed that the sequencing results were again ambiguous due to band doubling (Supplementary Fig. 7a), similar to the phosphorothioate sequencing results in the natural system. We treated the 2-iodoethanol-cleaved l-DNA fragments with natural CIP, and unexpectedly, the natural CIP treatment substantially improved the l-DNA sequencing results (Supplementary Fig. 7b), likely through the removal of 3′-monophosphates in l-DNAs through a previously unknown cross-chiral dephosphorylation activity of CIP as revealed by matrix-assisted laser desorption ionization–time of flight–mass spectrometry (MALDI–TOF–MS) (Supplementary Fig. 8). Hence, the sequence of band l-9-1 was readily determined (Fig. 1e and Supplementary Fig. 7b).

Additionally, we sequenced band l-9-2 using the phosphorothioate approach and observed that even with treatment by natural CIP, three nucleotide positions in the randomized region of the sequenced aptamer caused ambiguous reading (likely due to contaminating sequences) and resulted in eight most probable l-DNA aptamer sequences (Fig. 1f, Supplementary Fig. 7c and Supplementary Table 1). We reasoned that the incorrect sequences could be ruled out using DGGE by comparing the migration of potential aptamer sequences, since the correct sequence(s) should comigrate with band l-9-2 for the identical melting temperature (Supplementary Fig. 9a). Thus, we screened the natural versions (to save cost and the mirror-image enzymes) of the eight most probable l-DNA aptamer sequences in band l-9-2 (d-l-9-2-1 to d-l-9-2-8, Supplementary Table 1) by DGGE, to rule out the incorrect sequences. We observed that only the d-l-9-2-7 sequence comigrated with band l-9-2 (Supplementary Fig. 9b), suggesting that d-l-9-2-7 and band l-9-2 likely share the same sequence. Hence, the sequence of band l-9-2 was determined through a combination of a first DGGE to isolate (Fig. 1d), l-DNA sequencing-by-synthesis using the phosphorothioate approach (Fig. 1f) and a second DGGE to rule out the incorrect sequences (Supplementary Fig. 9b).

### Characterizing the selected l-DNA aptamers

**Fig. 2:**
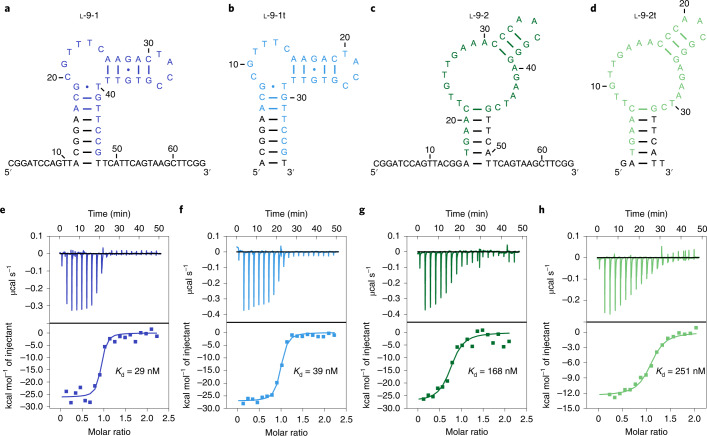
Characterizing the selected l-DNA aptamers. **a**–**d**, Secondary structures of the l-9-1 (**a**), l-9-1t (**b**, truncated version), l-9-2 (**c**) and l-9-2t (**d**, truncated version) aptamers predicted by Mfold, with nucleotides derived from the randomized region shown in blue, cyan, green and light green, respectively. **e**–**h**, ITC analysis of the l-9-1 (**e**), l-9-1t (**f**), l-9-2 (**g**) and l-9-2t (**h**) aptamers binding with native human thrombin, with *K*_d_ measured at 29, 39, 168 and 251 nM, respectively. The experiments were performed twice with similar results.

**Extended Data Fig. 2:**
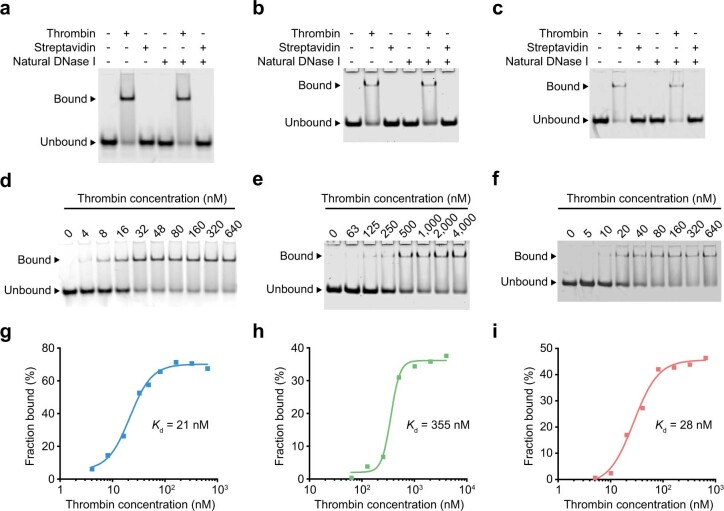
Characterizing the selected l-DNA aptamers. **a**-**c**, EMSA of 200 nM Cy5-l-9-1t (**a**), 200 nM Cy5-l-9-2t (**b**), or 200 nM Cy5-l-13t (**c**) aptamers binding with 1 μM native human thrombin or 1 μM streptavidin, without or with 50 units ml^-1^ natural DNase I, analyzed by 8% native PAGE (**a** and **c**) or 10% native PAGE with 5% (v/v) glycerol (**b**), and scanned by the Amersham Typhoon Biomolecular Imager under the Cy5 mode. The experiments were performed twice with similar results. **d**-**f**, EMSA of 35 nM Cy5-l-9-1t (**d**), 200 nM Cy5-l-9-2t (**e**), or 25 nM Cy5-l-13t (**f**) aptamers binding with various concentrations of native human thrombin, analyzed by 8% native PAGE (**d** and **f**), or 10% native PAGE with 5% (v/v) glycerol (**e**), and scanned by the Amersham Typhoon Biomolecular Imager under the Cy5 mode. The experiments were performed twice with similar results. **g**-**i**, Gel quantitation results of (**d**-**f**), with thrombin concentration shown in logarithmic scale and the fraction bound determined by the ImageJ software using the band intensity of the bound l-DNA aptamers relative to the total lane fluorescence intensity, and *K*_d_ measured at 21 nM (**g**, Cy5-l-9-1t), 355 nM (**h**, Cy5-l-9-2t), and 28 nM (**i**, Cy5-l-13t), respectively.

To measure the binding affinity of the sequenced l-DNA aptamers with native human thrombin, we prepared the l-9-1 and l-9-2 aptamers by solid-phase oligo synthesis (Fig. 2a,c), which bound native human thrombin with *K*_d_ measured by ITC in physiological buffer at 29 and 168 nM, respectively (Fig. 2e,g). Based on their secondary structures predicted by Mfold, we truncated the l-9-1 aptamer from 65 to 36 nucleotides (nt) and the l-9-2 aptamer from 65 to 38 nt (Fig. 2b,d), and observed that the truncated l-9-1t and l-9-2t aptamers bound native human thrombin with only slightly reduced binding affinity, with *K*_d_ measured by ITC at 39 and 251 nM, respectively (Fig. 2f,h). Meanwhile, binding was not detected between the l-9-1t and l-9-2t aptamers with streptavidin (Supplementary Figs. 10a,d and 11a,c), and the natural versions of the l-9-1t and l-9-2t aptamers (d-l-9-1t and d-l-9-2t) with native human thrombin (Supplementary Figs. 10b,e and 11b,d), suggesting that the binding between the l-9-1t and l-9-2t aptamers with native human thrombin was both target- and chiral-specific. Further shortening the l-9-1t aptamer from 36 to 32 nt led to roughly threefold reduction in binding affinity (*K*_d_ = 111 nM, Supplementary Fig. 10c,f), likely due to destabilization of the aptamer structure. Furthermore, the 5′-cyanine 5 (Cy5)-labeled l-9-1t and l-9-2t (Cy5-l-9-1t and Cy5-l-9-2t) aptamers formed stable complexes with native human thrombin as revealed by EMSA, which were, as expected, resistant to natural DNase I digestion (Extended Data Fig. 2a,b), with *K*_d_ measured by EMSA at 21 and 355 nM, respectively (Extended Data Fig. 2d,e,g,h).

To further evaluate the target-specificity of the l-DNA aptamers, we measured the binding affinity of the l-9-1t and l-9-2t aptamers with native bovine thrombin, which displays roughly 85% sequence identity with native human thrombin^28^. We observed that the l-9-1t and l-9-2t aptamers bound native bovine thrombin with *K*_d_ measured by ITC at 1,027 and 426 nM, respectively (Supplementary Fig. 12a,b), exhibiting roughly 26- and 1.7-fold reduction in binding affinity compared with native human thrombin, respectively, suggesting that the l-9-1t aptamer binds native human much tighter than with native bovine thrombin, while the l-9-2t aptamer binds both with similar affinities.

### Secondary selection and optimization of l-DNA aptamers from a partially randomized l-DNA library

The initial mirror-image selection gave rise to an l-DNA aptamer with high affinity (l-9-1) and another with moderate affinity (l-9-2). The suboptimal binding of the l-9-2 aptamer with native human thrombin (with *K*_d_ measured by ITC at 168 nM) prompted us to further optimize the l-DNA aptamer for improved binding affinity. The ability to optimize existing l-DNA aptamers through secondary selection is important for pharmaceutical and other clinical applications since the initially selected lead sequence often needs to be modified and improved to meet various practical needs.

**Fig. 3:**
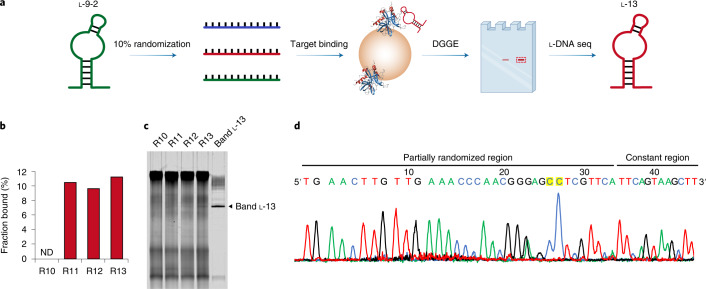
Secondary selection and optimization of l-DNA aptamers from a partially randomized l-DNA library. **a**, Schematic overview of the secondary selection and optimization of l-DNA aptamers from a partially randomized l-DNA library, with partial randomization of 34 nt at a frequency of 10% based on the l-9-2 aptamer. **b**, Monitoring the progress of secondary selection by EMSA, with the corresponding native PAGE shown in Extended Data Fig. 1d and the fraction bound determined by the ImageJ software using the band intensity of the bound l-DNA pool relative to the total lane fluorescence intensity. ND, (binding) not detected. The experiment was performed twice with similar results. **c**, DGGE analysis of the corresponding l-DNA library and pools, as well as the isolated band l-13, re-amplified by mirror-image PCR using d-Dpo4-5m with l-dNTPs and l-DNA primers, analyzed by 10% denaturing PAGE in 2.1–4.2 M urea and 12–24% formamide, stained by SYBR Green II and scanned by the Amersham Typhoon Biomolecular Imager under the Cy2 mode. The experiment was performed twice with similar results. **d**, Sequencing chromatogram of band l-13 amplified by mirror-image PCR using d-Dpo4-5m with l-dNTPαSs and 5′-FAM-labeled l-DNA forward sequencing primer after natural CIP treatment, with the two mutations highlighted in yellow and the corresponding sequencing gel shown in Supplementary Fig. 14. The experiment was performed twice with similar results.

**Fig. 4:**
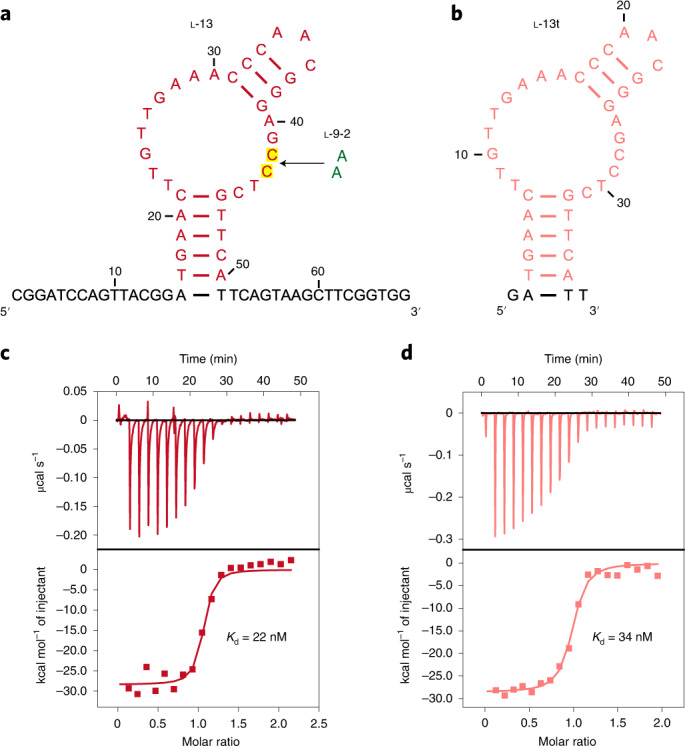
Characterizing the optimized l-DNA aptamers from secondary selection. **a**,**b**, Secondary structures of the l-13 (**a**) and l-13t (**b**, truncated version) aptamers predicted by Mfold, with nucleotides derived from the secondary selection shown in red and pink, respectively, and the two mutations (both from A to C) highlighted in yellow. **c**,**d**, ITC analysis of the l-13 (**c**) and l-13t (**d**) aptamers binding with native human thrombin, with *K*_d_ measured at 22 and 34 nM, respectively. The experiments were performed twice with similar results.

For the secondary selection and optimization of the l-9-2 aptamer, we first generated a partially randomized l-DNA library of roughly 1 × 10^11^ distinct sequences by solid-phase oligo synthesis, with partial randomization of a 34-nt stem-loop region at a frequency of 10% based on the l-9-2 aptamer, flanked by two constant regions for primer binding (Extended Data Fig. 1c). Next, we performed mirror-image selection of the partially randomized l-DNA library targeting native human thrombin (Fig. 3a). After three rounds of enrichment and mirror-image PCR amplification (Fig. 3b, Extended Data Fig. 1d and Supplementary Fig. 13), we applied DGGE to isolate a single band (l-13) from R13, accounting for roughly 0.2% of the total lane fluorescence intensity of R13 (Fig. 3c). We amplified band l-13 by mirror-image PCR using d-Dpo4-5m with l-dNTPs and l-DNA primers, and analyzed the mirror-image PCR product by another DGGE, revealing a predominant band accounting for roughly 13% of the total lane fluorescence intensity (Fig. 3c). We applied l-DNA sequencing-by-synthesis using the phosphorothioate approach to determine the enriched l-DNA aptamer sequence (Fig. 3d and Supplementary Fig. 14), and successfully identified a mutant sequence of the l-9-2 aptamer with two adenosines (A42 and A43) mutated to cytidines (C42 and C43) in the partially randomized region (Figs. 3d and 4a).

### Characterizing the optimized l-DNA aptamers from secondary selection

We show that the A42C and A43C mutations are located in the loop region of the optimized l-13 aptamer based on its secondary structure predicted by Mfold (Fig. 4a), and that the l-13 aptamer bound native human thrombin with *K*_d_ measured by ITC in physiological buffer at 22 nM (Fig. 4c), exhibiting roughly eightfold improvement of binding affinity with native human thrombin compared with the parental l-9-2 aptamer. We also truncated the l-13 aptamer from 68 to 38 nt based on its secondary structure predicted by Mfold (Fig. 4b), and observed that the truncated l-13t aptamer bound native human thrombin with only slightly reduced binding affinity (*K*_d_ = 34 nM, Fig. 4d). Additionally, we show that the 5′-Cy5-labeled l-13t (Cy5-l-13t) aptamer formed stable complexes with native human thrombin with *K*_d_ measured by EMSA at 28 nM (Extended Data Fig. 2c,f,i). We further show that despite having roughly sevenfold higher binding affinity with native human thrombin than the l-9-2t aptamer, the binding affinity of l-13t aptamer with native bovine thrombin was much lower (with *K*_d_ measured by ITC at 340 nM, Supplementary Fig. 12c), similar to that of the l-9-2t aptamer. These results indicate that with only two mutations in the partially randomized loop region, both the binding affinity and target-specificity of the l-DNA aptamer were substantially improved.

### l-DNA aptamer thrombin inhibitors

**Fig. 5:**
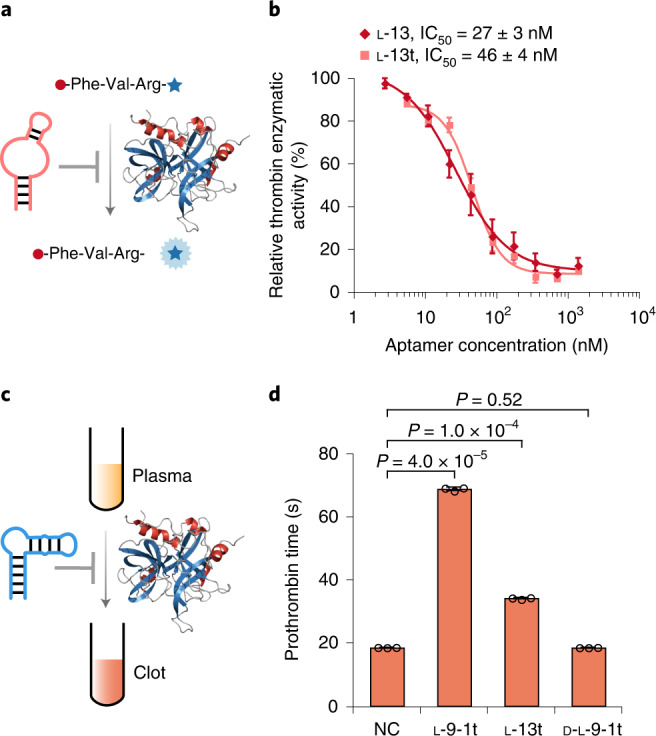
l-DNA aptamer thrombin inhibitors. **a**, Schematic overview of inhibiting native human thrombin enzymatic activity with the selected l-DNA aptamers, native human thrombin and a fluorogenic substrate, benzoyl-Phe-Val-Arg-AMC. **b**, Relative thrombin enzymatic activity of 10 nM native human thrombin and 100 μM benzoyl-Phe-Val-Arg-AMC, incubated with the l-13 and l-13t aptamers in physiological buffer, with IC_50_ measured at 27 ± 3 and 46 ± 4 nM, respectively. Data are presented as mean ± s.d. (*n* = 3, independent measurements) with aptamer concentration shown in logarithmic scale. **c**, Schematic overview of the in vitro coagulation assay with the selected l-DNA aptamers and human plasma. **d**, Prothrombin time of 50% (v/v) human plasma incubated with 2.5 μM l-9-1t or l-13t aptamer, or the natural version of the l-9-1t aptamer (d-l-9-1t). NC, negative control with 50% human plasma in physiological buffer alone. *P* values are calculated from Student’s two-tailed *t*-test. Data are presented as mean ± s.d. (*n* = 3, independent measurements).

Next, we tested the inhibition of thrombin enzymatic activity by the l-9-1, l-9-2 and l-13 aptamers in physiological buffer with 100 μM benzoyl-Phe-Val-Arg-7-amino-4-methylcoumarin (AMC) (Fig. 5a and Supplementary Fig. 15a–c), a fluorogenic substrate for thrombin^29^. We observed that the l-9-2 and l-13 aptamers inhibited thrombin enzymatic activity with half-maximum inhibitory concentrations (IC_50_) measured at 317 ± 128 and 27 ± 3 nM, respectively (Fig. 5b and Supplementary Fig. 16), largely consistent with their *K*_d_ measured by ITC (168 and 22 nM, respectively). In comparison, the l-DNA libraries before selections did not inhibit thrombin enzymatic activity at concentrations of up to 8 μM (Supplementary Fig. 15d,e). We also tested the inhibition of thrombin enzymatic activity by the truncated l-9-2t and l-13t aptamers and measured slightly higher IC_50_ of 479 ± 65 and 46 ± 4 nM, respectively (Fig. 5b and Supplementary Figs. 15f,g and 16), largely consistent with their *K*_d_ measured by ITC (251 and 34 nM, respectively). However, the l-9-1 and l-9-1t aptamers did not inhibit thrombin enzymatic activity at concentrations of up to 8 μM (Supplementary Fig. 15a,h), suggesting different binding sites of native human thrombin targeted by the l-DNA aptamers. In comparison, the natural versions of the l-9-2t and l-13t aptamers (d-l-9-1t and d-l-13t) did not inhibit thrombin enzymatic activity at concentrations of up to 8 μM (Supplementary Fig. 15i,j).

As a more realistic demonstration of the therapeutic potential of the selected l-DNA aptamers, we performed an in vitro coagulation assay on human plasma (Fig. 5c). We observed that with the addition of 2.5 μM l-9-1t and l-13t aptamers, the prothrombin time of human plasma measured roughly four- and twofold longer than those of the controls without l-DNA aptamers or with the natural version of the l-9-1t aptamer (d-l-9-1t), respectively (Fig. 5d). Since prothrombin time is inversely correlated with thrombin activity in a nonlinear model^30^, these results indicate that both l-DNA aptamers substantially inhibited thrombin activity, potentially with the l-9-1t aptamer blocking one of the exosites of native human thrombin and the l-13t aptamer blocking the active site.

### l-DNA aptamer sensor

**Fig. 6:**
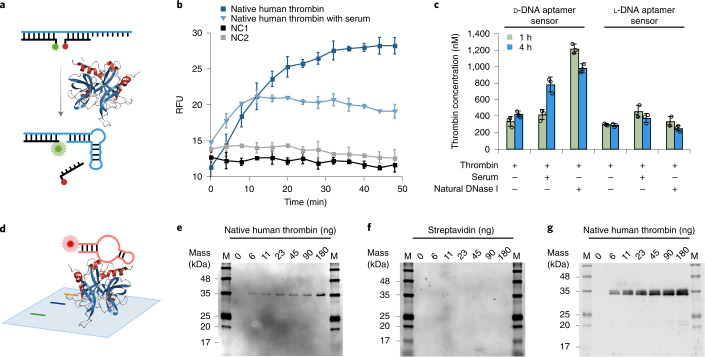
l-DNA aptamer sensor and western blot. **a**, Schematic overview of detecting native human thrombin with the l-DNA aptamer sensor, by hybridizing the l-9-1t aptamer strand (cyan) with an l-DNA fluorophore strand labeled with FAM (green) and an l-DNA quencher strand labeled with DABCYL (red). **b**, Measured relative fluorescence of the l-DNA aptamer sensor incubated with 1 μM native human thrombin in physiological buffer alone, or physiological buffer with 10% human serum for up to 48 min, with excitation wavelength at 494 nm and emission wavelength at 518 nm, and measurements taken every 4 min. NC1, negative control with the l-DNA aptamer sensor in physiological buffer alone. NC2, negative control with the l-DNA aptamer sensor in physiological buffer with 10% human serum. RFU, relative fluorescence unit. Data are presented as mean ± s.d. (*n* = 3, independent measurements). **c**, Measured thrombin concentrations by the d- and l-DNA aptamer sensors with 300 nM native human thrombin in physiological buffer alone, or in physiological buffer with 10% human serum or 50 units ml^-1^ natural DNase I, incubated for up to 4 h. Data are presented as mean ± s.d. (*n* = 3, independent measurements), with the corresponding data shown in Supplementary Table 2. **d**, Schematic overview of detecting native human thrombin immobilized on a nitrocellulose membrane with l-DNA aptamer western blot. **e**–**g**, Native human thrombin (**e** and **g**) or streptavidin (**f**) separated by 15% SDS–PAGE, transferred to a nitrocellulose membrane, incubated with 500 nM Cy5-l-13t aptamer (**e** and **f**) or mouse monoclonal primary antibody targeting native human thrombin and Alexa Fluor 647-labeled goat anti-mouse IgG polyclonal secondary antibody (**g**) and scanned by the Amersham Typhoon Biomolecular Imager under the Cy5 mode. M, prestained protein marker labeled with fluorescent dye. The experiments were performed twice with similar results.

To further demonstrate the potential practical applications of the thrombin-binding l-DNA aptamers, we designed a structure-switching l-DNA aptamer sensor based on previous designs^31^, by hybridizing the high-affinity l-9-1t aptamer with an l-DNA fluorophore strand with 5′-labeled fluorescein (FAM) and an l-DNA quencher strand with 3′-labeled 4-((4-(dimethylamino)phenyl)azo)benzoic acid (DABCYL) (Fig. 6a). Upon binding native human thrombin, the l-DNA aptamer sensor undergoes structure switching, releasing the quencher strand and leading to increases of relative fluorescence with linear response in the range of roughly 125–1,000 nM (Fig. 6b and Supplementary Fig. 17a). In comparison, the l-DNA aptamer sensor did not respond to the addition of 1 μM streptavidin or 1 μM native bovine thrombin (Supplementary Fig. 17b), consistent with the ITC results (Supplementary Figs. 10d and 12a). To evaluate the influence of serum enzymes on the biostability and thrombin-sensing ability of the l-DNA aptamer sensor, we incubated the l-DNA aptamer sensor in physiological buffer with 10% (v/v) human serum, which provided a physiologically relevant nuclease-rich environment (although l-DNA aptamer sensor may also work with undiluted serum, it requires more sophisticated design to avoid background autofluorescence from undiluted serum^32^). We observed that the l-DNA aptamer sensor responded to the addition of native human thrombin in physiological buffer with 10% human serum with linear response in the range of roughly 250–2,000 nM (Fig. 6b and Supplementary Fig. 17a). In parallel, we also designed a d-DNA aptamer sensor based on the d-DNA aptamer d-6 (Supplementary Fig. 18). We applied both the d- and l-DNA aptamer sensors to detect 300 nM native human thrombin in physiological buffer, with 10% human serum or with 50 units ml^−1^ natural DNase I (one of the major nucleases in serum^33^) for up to 4 h, and observed that the thrombin concentrations measured by the l-DNA aptamer sensor were generally accurate, whereas those by the d-DNA aptamer sensor were roughly two- to fourfold higher (Fig. 6c and Supplementary Table 2). We attribute the erroneous measurements by the d-DNA aptamer sensor but not l-DNA aptamer sensor to the increases of relative fluorescence resulting from degradation of the d-DNA aptamer sensor by the serum enzymes and natural DNase I, largely consistent with the estimated half-life (*t*_1/2_) of 1.7 ± 0.1 h for the d-DNA aptamer sensor incubated in physiological buffer with 10% human serum as measured by denaturing PAGE (Supplementary Fig. 19a,b), causing premature release of the FAM fluorophore and DABCYL quencher. To further validate the biostability of the l-DNA aptamer sensor, we incubated the sensor in physiological buffer with 83% human serum, and observed no notable degradation of the l-DNA aptamer strand by denaturing PAGE for up to 30 d (720 h), whereas the d-DNA aptamer strand was rapidly degraded with an estimated *t*_1/2_ of 2.1 ± 0.4 h (Supplementary Fig. 19c–f), largely consistent with the results from previous studies on other d-DNA aptamers in human serum^6,34^.

### l-DNA aptamer western blot

Moreover, we applied the Cy5-l-13t aptamer to a proof-of-concept l-DNA aptamer western blot experiment for detecting native human thrombin immobilized on a nitrocellulose membrane (Fig. 6d). We analyzed 6–180 ng native human thrombin or streptavidin by sodium dodecyl sulphate (SDS)–PAGE, which was subsequently transferred to a nitrocellulose membrane and incubated with 500 nM Cy5-l-13t aptamer at room temperature for 1 h. We detected fluorescent bands consistent with the molecular mass of native human thrombin (roughly 36 kDa) with detection limit below 6 ng (Fig. 6e), whereas no band at the expected molecular mass of streptavidin (roughly 18 kDa) was detected (Fig. 6f). In comparison, we analyzed 6–180 ng native human thrombin by traditional western blot using mouse monoclonal primary antibody targeting native human thrombin and Alexa Fluor 647-labeled (with similar excitation and emission wavelengths to Cy5) goat anti-mouse IgG polyclonal secondary antibody, and detected fluorescent bands consistent with the molecular mass of native human thrombin (roughly 36 kDa) (Fig. 6g). Further exploration of basic research tools based on l-DNA aptamers may open new doors of opportunity for practical applications thanks to their exceptional biostability during storage and use, and convenience of production by solid-phase oligo synthesis compared with traditional antibody-based tools.

## Discussion

In this work, we developed a mirror-image selection scheme for the directed evolution and selection of biostable l-DNA aptamers from large randomized l-DNA libraries, with several l-DNA aptamers targeting different binding sites of native human thrombin obtained, leading to various applications. Despite being a young method, in that we have only tested it on one of many possible target molecules as the first example, mirror-image selection presents a generalizable scheme that can be applied to virtually all complex biological targets including natural proteins (native or recombinant) translated and folded in vivo, many of which are with native PTMs. Moreover, almost infinitely complex molecules and even cells and tissues^35,36^ can be targeted with a relatively limited set of mirror-image molecular tools, to discover binders, inhibitors and biomarkers without requiring extensive knowledge of the selection target itself. The biostability of the l-DNA pools and the mirror-image molecular tools will also make the system entirely resistant to degradation by nucleases, which is particularly important for selections targeting low-purity molecules, cells and tissues, and even nucleases themselves. Similar selection schemes may also find applications in the discovery of l-RNA aptamers, l-ribozymes and l-DNAzymes that interact with and catalyze the manipulation (for example, ligation, cleavage and modification and so on) of biologically important proteins and RNA structures^9^^,10–12,37^, especially if the efficiency and fidelity of the current mirror-image transcription and reverse transcription system can be improved further^38^. The directed evolution and selection of mirror-image peptide sequences may be also achieved in the future, although it would require a mirror-image translation system based on a functional mirror-image ribosome that is yet to be synthesized^39–43^, with which one can perform the mirror-image version of ribosome or messenger RNA (mRNA) display^44,45^.

The unique ability of mirror-image aptamers to evade biodegradation during storage and use, and the convenience of production by automated oligo synthesizers will make them suitable for various applications traditionally carried out by natural aptamers and antibodies. For example, drug-conjugated mirror-image aptamers may be developed into biostable nanocarriers for targeted delivery of chemotherapeutic, small interfering RNA and microRNA drugs^46^. Other potential applications of biostable mirror-image aptamers in physiologically relevant nuclease-rich environments may include, but are not limited to: enzyme-linked immunosorbent assay (ELISA), immunohistochemistry, flow cytometry and molecular imaging both in vitro and in vivo^47^. Additionally, mirror-image aptamers may be developed into biostable chromatography resins for the separation of racemic mixtures of small-molecule compounds^48^. The versatility of the mirror-image selection scheme and its convenience of directly using virtually all native protein targets for selection will also make it well-suited for the quick-turnaround discovery of biostable mirror-image aptamers targeting emerging pathogens for diagnostic and therapeutic purposes^49^.

The use of relatively accessible mirror-image molecular tools in the current mirror-image selection scheme, such as the small thermostable mirror-image DNA polymerase (d-Dpo4-5m) for library amplification and l-DNA sequencing-by-synthesis, will make it practical for large-scale academic and industrial applications. Nonetheless, the error-prone nature and suboptimal amplification efficiencies of d-Dpo4-5m may cause inaccurate and inefficient amplification of rare sequences under more stringent selection conditions, particularly with randomized sequences substantially longer than 30–40 nt. The use of another mirror-image DNA polymerase in conjunction, such as the high-fidelity mirror-image *Pfu* DNA polymerase for high-fidelity mirror-image PCR^14^ may help to address this issue. Another major technical hurdle to overcome is the throughput of l-DNA sequencing. Although by repurposing DGGE, mirror-image selection can conclude without requiring the l-DNA pools to fully converge, DGGE isolation of near-single sequences may not always be achieved, especially for sequences with similar melting temperatures. Nonetheless, despite being constrained by many technical hurdles at the moment, more advanced mirror-image molecular tools will continue to be developed to help realize more accessible and versatile mirror-image selection schemes. For example, the development of high-throughput l-DNA sequencing methods by adopting the nanopore sequencing or massively parallel sequencing-by-synthesis techniques^50^, may achieve better sequence coverage of the selected l-DNA aptamers, reduce the number of mirror-image selection rounds and make mirror-image selection more practical for future applications.

## Methods

### Materials

All the l-DNA oligos were synthesized on the H-8 oligo synthesizer (K&A Laborgeraete). All the d-DNA oligos were ordered from Genewiz. l-deoxynucleoside phosphoramidites were purchased from ChemGenes. Hexaethylene glycol spacer (Sp18) phosphoramide was purchased from Glen Research. FAM and Cy5 phosphoramides, as well as DABCYL and phosphatecontrolled pore glass (CPG), were purchased from Ruibiotech. All the d- and l-DNA oligos were purified by high-performance liquid chromatography (HPLC) or denaturing PAGE before use, with their sequences listed in Supplementary Tables 1 and 6. l-dNTPs and l-dNTPαSs were synthesized from l-deoxynucleosides (ChemGenes)^51^. d-dNTPs and d-dNTPαSs were purchased from TriLink Biotechnologies. l-Dpo4-5m with an N-terminal His_6_ tag was expressed in *Escherichia coli* strain BL21 and purified as described in the literature^15^. d-Dpo4-5m was synthesized and folded as described in the literature^15,16^, except that automated peptide synthesizers were used and norleucine (Nle) was replaced by methionine (Met). The FastPfu Fly DNA polymerase was purchased from TransGen Biotech. 2-iodoethanol was purchased from Aladdin Bio-Chem Technology Co., Ltd. Native human α-thrombin and native bovine α-thrombin of plasma origin were purchased from Haematologic Technologies. Streptavidin, natural CIP and natural DNase I were purchased from New England Biolabs. Prestained protein marker labeled with fluorescent dye was purchased from Solarbio Life Sciences. Human serum was purchased from ZhongKeChenYu Biotech. Human plasma was obtained with written informed consent from a 33-year-old healthy male volunteer following the protocol approved by the Institution Review Board of Tsinghua University (project no. 20210173). Mouse monoclonal primary antibody targeting native human thrombin and Alexa Fluor 647-labeled goat anti-mouse IgG polyclonal secondary antibody were purchased from Abcam. ExRed was purchased from Beijing Zoman Biotech. *N*-hydroxysuccinimide(NHS)-activated magnetic beads and SYBR Green II were purchased from Thermo Fisher Scientific. Benzoyl-Phe-Val-Arg-AMC was purchased from Sigma-Aldrich.

### l-DNA library preparation

The 30-nt randomized region of the d- or l-DNA library with 65 nt in total length was synthesized with molar concentration ratios of d- or l- dA, dC, dG, dT phosphoramidites at 30:25:23:20 to achieve approximately equal coupling efficiencies^52^. PAGE purification was performed to remove the aggregation-prone DNA as described in the literature^53^. Briefly, 5 nmol synthetic d- or l-DNA library was loaded on slabs of 1 × 200 × 550 mm, separated by PAGE composed of a denaturing top section (1 × 200 × 50 mm) containing 7 M urea and 8% acrylamide in 0.5× Tris-borate-EDTA (TBE), and a nondenaturing bottom section (1 × 200 × 500 mm) containing 10% acrylamide and 10 mM Mg(OAc)_2_ in 0.5× TBE. The gel was run at 10 W (constant power) for 6 h and stained by SYBR Green II. The fastest-migrating third of the band was isolated and purified by the ‘crush and soak’ method^54^. Approximately 165 pmol of native PAGE-purified library (of roughly 1 × 10^14^ distinct sequences) was amplified by natural or mirror-image PCR using l- or d-Dpo4-5m with d- or l-dNTPs and d- or l-DNA primers, in which the reverse primer contained a poly d(A)_20_ tail modified by Sp18 to generate PCR product with strands of different lengths for strand separation by denaturing PAGE^55^ (Fig. 1b). The natural and mirror-image PCR program settings were 86 °C for 3 min (initial denaturation); 86 °C for 30 s, 50 °C for 1 min and 65 °C for 2 min, for 15 cycles; 65 °C for 5 min (final extension). The 65-nt forward strand was separated from the 85-nt Sp18-modified reverse strand by 10% denaturing PAGE in 7 M urea and used as the starting d- or l-DNA library for selection.

### Mirror-image selection of d- or l-DNA aptamers targeting native human thrombin

Magnetic beads coupled with native human thrombin were prepared from NHS-activated magnetic beads according to the manufacturer’s instructions (Thermo Fisher Scientific). Briefly, 300 μl of native human thrombin at a concentration of 0.1 mg ml^−1^ was mixed with 3 mg of NHS-activated magnetic beads in coupling buffer (20 mM HEPES-NaOH, 150 mM NaCl, 5% (v/v) glycerol, pH 7.4). The coupling reaction was performed at room temperature for 2 h, before being quenched by 3 M ethanolamine at pH 9.0. After coupling, the beads were resuspended in 300 μl of selection buffer (20 mM HEPES-NaOH, 150 mM NaCl, 5 mM KCl, 2 mM MgCl_2_, 1 mM CaCl_2_, 0.05% (v/v) Tween 20, pH 7.4). For R1, roughly 600 pmol d- or l-DNA library (both of roughly 1 × 10^14^ distinct sequences) in a total volume of 250 μl was heated to 85 °C for 5 min in selection buffer and slowly cooled to 25 °C over 10 min for annealing, after which 50 μl of protein-free NHS-activated magnetic beads were added and the mixture was incubated under gentle rotation at room temperature for 1 h. In each selection round, a negative selection step against 50 μl of protein-free NHS-activated magnetic beads was performed. The supernatant was mixed with 100 μl of magnetic beads coupled with native human thrombin in a total volume of 400 μl and incubated under gentle rotation at room temperature for 1 h, after which the beads were separated from the supernatant by a DynaMag-2 magnet (Thermo Fisher Scientific) and briefly washed three times (10 s per wash) with 400 μl of selection buffer. The bound DNA was eluted from the beads by 25 mM NaOH and 5 mM EDTA, and precipitated by ethanol. The recovered d- or l-DNA was used as template for natural or mirror-image PCR using l- or d-Dpo4-5m with d- or l-dNTPs and d- or l-DNA primers to generate the d- or l-DNA pool for the next round. The number of natural or mirror-image PCR cycles for each selection round was determined based on the result of 10 μl-scale PCR (Supplementary Fig. 1a,b). As shown in Supplementary Tables 3 and 4, the amount of DNA pool gradually decreased from roughly 600 pmol in R1 to roughly 50 pmol in R6 (for d-DNA pools), and from roughly 600 pmol in R1 to roughly 30 pmol in R9 (for l-DNA pools), respectively. The volume of magnetic beads coupled with native human thrombin gradually decreased from 100 μl in R1 to 10 μl in R6 (for d-DNA pools), and from 100 μl in R1 to 3 μl in R9 (for l-DNA pools), respectively. The wash step gradually increased from three 10 s washes in R1 to six 10-min washes in R6 (for d-DNA pools) and from three 10 s washes in R1 to eight 10-min washes in R9 (for l-DNA pools), respectively.

### EMSA

The d- or l-DNA pools and d- or l-DNA aptamers were heated to 85 °C for 5 min in selection buffer and slowly cooled to 25 °C over 10 min for annealing, before being mixed with native human thrombin or streptavidin in selection buffer with 10% (v/v) glycerol. The mixtures were incubated at room temperature for 30 min, and analyzed by 8% n﻿ative PAGE in 1× running buffer (20 mM HEPES-NaOH, 50 mM NaOAc, 5 mM KOAc, 2 mM Mg(OAc)_2_, 1 mM CaCl_2_, pH 7.4 (for the d- or l-DNA pools, the d-6, Cy5-l-9-1t and Cy5-l-13t aptamers)), or by 10% native PAGE in 1× running buffer with 5% (v/v) glycerol added to both the gel and running buffer (for the Cy5-l-9-2t aptamer) as glycerol enhances the stability of weakly interacting protein–nucleic acid complexes and prevents their dissociation in EMSA^56^. The gel was run at 150 V (constant voltage) at 4 °C for 1–2 h, stained by SYBR Green II and scanned by the Amersham Typhoon Biomolecular Imager (Cytiva) under the Cy2 mode (for d- or l-DNA pools and the d-6 aptamer), or without staining under the Cy5 mode (for the Cy5-labeled l-DNA aptamers). Gel quantitation was performed by the ImageJ software (https://imagej.nih.gov/ij), with *K*_d_ calculated by fitting the fraction bound to the sigmoidal equation using the KaleidaGraph software (Synergy Software).

### DGGE

The d- or l-DNA pools, and d- or l-DNA aptamers were amplified by natural or mirror-image PCR using l- or d-Dpo4-5m with d- or l-dNTPs and d- or l-DNA primers, with the forward primer containing a GC-rich region (GC-clamp) to prevent the double-stranded PCR products from complete melting during DGGE^57^ (Supplementary Fig. 3a). The natural or mirror-image PCR products were purified by 3% sieving agarose gel electrophoresis and mixed with 2× loading buffer (100 mM Tris-HCl, 10 mM EDTA, 30% glycerol, pH 7.0), and separated by 7.5% polyacrylamide gel (for d-DNA pools) or 10% polyacrylamide gel (for l-DNA pools) composed of a linear denaturant gradient from 2.1 M urea, 12% (v/v) formamide (top) to 4.2 M urea and 24% (v/v) formamide (bottom) in 1× Tris-acetate-EDTA (TAE). The gel was run at 100 V at 60 °C (constant temperature) for 6 h (for d-DNA pools) or at 75 V at 60 °C for 13 h (for l-DNA pools). For DGGE isolation of d- or l-DNA aptamer sequences, 500 ng of natural or mirror-image PCR products were separated by DGGE, stained by SYBR Green II, isolated by cutting the gel on the eBL-100 transilluminator (Eastwin), purified by the ‘crush and soak’ method^54^, and re-amplified by natural or mirror-image PCR using l- or d-Dpo4-5m with d- or l-dNTPs and d- or l-DNA primers. To rule out the incorrect sequences from the band l-9-2 sequencing results, the natural versions of the eight most probable l-DNA aptamer sequences in band l-9-2 (d-l-9-2-1 to d-l-9-2-8, Supplementary Table 1) were amplified by natural PCR using the FastPfu Fly DNA polymerase with d-dNTPs and d-DNA primers, separated by DGGE, stained by SYBR Green II and scanned by the Amersham Typhoon Biomolecular Imager under the Cy2 mode. The melting temperatures (*T*_m_) were calculated by OligoCalc^58^ using default parameters of the nearest-neighbor thermodynamic model.

### High-throughput sequencing of the selected d-DNA aptamers

The R6 d-DNA pool and band d-6 isolated by DGGE were amplified by natural PCR using l-Dpo4-5m with d-dNTPs and d-DNA primers. The PCR products were purified by 2.5% agarose, and sequenced on the Illumina HiSeq X Ten platform. The raw Illumina reads were processed and sorted by percent abundance using the Galaxy server (https://usegalaxy.org).

### MALDI–TOF–MS

MALDI–TOF–MS was used to analyze the dephosphorylation of l-DNAs by natural CIP. Approximately 100 ng of 3′-monophosphate-labeled l-DNA oligo was treated with 20 units of natural CIP, incubated in 1× CutSmart buffer (New England Biolabs) at 37 °C for 1 h, desalted by a C18 spin column (Thermo Fisher Scientific), and analyzed under the positive linear mode by the Applied Biosystems 4800 Plus MALDITOF/TOF Analyzer (Thermo Fisher Scientific).

### l-DNA aptamer sequencing

l-DNA aptamers isolated by DGGE were amplified by mirror-image PCR using d-Dpo4-5m in four separate reactions, in which one of the l-dNTPs was replaced by the corresponding l-dNTPαS (ref. ^14^), with 5′-FAM-labeled l-DNA forward sequencing primer and unlabeled l-DNA reverse primer listed in Supplementary Table 6. The 5′-FAM-labeled PCR products were purified by 10% denaturing PAGE in 7 M urea and dissolved in 10 mM Tris-HCl at pH 7.4 to a final concentration of roughly 20 ng μl^−1^. For each sequencing reaction, 5 μl of 5′-FAM-labeled l-DNA was mixed with 5 μl of cleavage solution containing 2% (v/v) 2-iodoethanol in ddH_2_O, before being heated to 95 °C for 3 min and quickly placed on ice. For the removal of 3′-monophosphate from the 2-iodoethanol-cleaved DNA fragments, each sequencing reaction was treated with 5 units of natural CIP, incubated in 1× CutSmart buffer at 37 °C for 1 h, before being mixed with 10 μl of 2× loading buffer containing 95% formamide and 10 mM EDTA. The samples were loaded on slabs of 0.4 × 340 × 300 mm, analyzed by 10% denaturing PAGE in 7 M urea as described in the literature^14^ and scanned by the Amersham Typhoon Biomolecular Imager under the Cy2 mode. Chromatogram analysis was performed by the ImageJ software and Microsoft Excel.

### ITC

Native human thrombin, native bovine thrombin and streptavidin in storage buffer were dialyzed against physiological buffer (20 mM HEPES-NaOH, 150 mM NaCl, 5 mM KCl, 2 mM MgCl_2_, 1 mM CaCl_2_, pH 7.4) at 4 °C for 16 h. Since previous studies suggested that incubation at temperatures such as 25 °C for over 24 h may cause thrombin autolysis^59^, we performed control experiments to rule out autolysis by separating native human and bovine thrombin before and after dialysis by 15% SDS–PAGE, stained by Coomassie brilliant blue, and scanned by the ChemiDoc XRS + system (Bio-Rad) (Supplementary Fig. 6a,b). d- and l-DNA aptamers were equilibrated in physiological buffer by ultrafiltration, before being heated to 85 °C for 5 min and slowly cooled to 25 °C over 10 min for annealing. ITC was performed using the MicroCal iTC_200_ Microcalorimeter (GE Healthcare) with 7–20 μM native human thrombin, native bovine thrombin or streptavidin in the reaction cell and 70–200 μM d- or l-DNA aptamer in the injection syringe under stirring at 750 r.p.m. at 25 °C. To measure the heat of dilution, 70–200 μM d- or l-DNA aptamer was added to physiological buffer in the absence of proteins. To further rule out potential thrombin autolysis that may affect the ITC results, we performed control experiments with physiological buffer alone added to 7 μM native human or bovine thrombin and detected no apparent heat flow at 25 °C for up to 1 h (Supplementary Fig. 6c,d). Data fitting was performed using the MicroCal Origin software (GE Healthcare).

### Secondary selection and optimization of l-DNA aptamers from a partially randomized l-DNA library

The partially randomized l-DNA library was synthesized by doping the 34-nt stem-loop region of the l-9-2 aptamer with the other three l-deoxynucleoside phosphoramidites at molar concentration ratios of 27:1:1:1 (Extended Data Fig. 1c and Supplementary Table 6), before being purified by 10% denaturing PAGE in 7 M urea. For R10, approximately 50 pmol of a partially randomized library (of roughly 1 × 10^11^ distinct sequences) was amplified by mirror-image PCR using d-Dpo4-5m with l-dNTPs and l-DNA primers, and purified by 10% denaturing PAGE in 7 M urea. The mirror-image PCR, strand separation, target binding, elution, DGGE and l-DNA sequencing experiments were performed using the methods described above with conditions listed in Supplementary Table 5.

### l-DNA aptamer thrombin enzymatic activity assay

l-DNA aptamers were heated to 85 °C for 5 min in physiological buffer and slowly cooled to 25 °C over 10 min for annealing, with native human thrombin added to a final concentration of 10 nM. The mixture was incubated in physiological buffer at room temperature for 30 min, followed by addition of 100 μM fluorogenic substrate, benzoyl-Phe-Val-Arg-AMC. Relative fluorescence was measured by the Varioskan Flash system (Thermo Fisher Scientific) with excitation wavelength at 350 nm and emission wavelength at 450 nm. Relative thrombin enzymatic activity was determined by change of relative fluorescence unit (ΔRFU), with relative fluorescence unit (RFU) measured at 0 min set to 0 and RFU of the negative control in physiological buffer alone set to 100%, and ΔRFU measured at 16 min was used to calculate the relative thrombin enzymatic activity (Supplementary Fig. 15a–j). IC_50_ was calculated by fitting the relative thrombin enzymatic activity to the sigmoidal equation using the KaleidaGraph software.

### l-DNA aptamer in vitro coagulation assay

The d- and l-DNA aptamers were heated to 85 °C for 5 min in 180 μl of physiological buffer, slowly cooled to 25 °C over 10 min for annealing and incubated with 180 μl of human plasma (to a final concentration of 2.5 μM for the d- and l-DNA aptamers) at room temperature for up to 10 min. The prothrombin time was measured by the STA R Max Coagulation Analyzer according to the manufacturer’s instructions (Stago).

### l-DNA aptamer sensor

The d- or l-DNA aptamer sensor containing 250 nM 5′-FAM-labeled fluorophore strand, 750 nM 3′-DABCYL-labeled quencher strand and 500 nM d-6 or l-9-1t aptamer strand (Fig. 6a) was incubated with 300 nM native human thrombin in physiological buffer alone or physiological buffer with 10% (v/v) human serum at 37 °C for up to 4 h. Relative fluorescence was measured by the Varioskan Flash system with excitation wavelength at 494 nm and emission wavelength at 518 nm. The standard curves were plotted using 0, 125, 250, 500 or 1,000 nM native human thrombin and relative fluorescence was measured after incubation in physiological buffer at 37 °C for 1 h (Supplementary Figs. 17a and 18c). ΔRFU was used for data fitting, with RFU measured with the d- or l-DNA aptamer sensor in physiological buffer alone set to 0. For measurements in physiological buffer with 10% (v/v) human serum, the standard curves were plotted using 0, 250, 500, 1,000 or 2,000 nM native human thrombin and relative fluorescence was measured after incubation in physiological buffer with 10% human serum at 37 °C for 1 h (Supplementary Figs. 17a and 18c). To evaluate the biostability of the d- and l-DNA aptamer sensors, the sensors were incubated in physiological buffer with 10% human serum at 37 °C for up to 24 h (for the d-DNA aptamer sensor) or in physiological buffer with 83% (v/v) human serum at 37 °C for up to 24 h (for the d-DNA aptamer sensor) or up to 30 d (720 h) (for the l-DNA aptamer sensor). Samples were mixed with 2× loading buffer containing 95% formamide and 10 mM EDTA, and quickly placed at −20 °C, analyzed by 10% denaturing PAGE in 7 M urea, and scanned by the Amersham Typhoon Biomolecular Imager under the Cy2 mode. Gel quantitation was performed by the ImageJ software, with the half-life (*t*_1/2_) calculated by fitting the relative band intensity to the one phase decay equation using the KaleidaGraph software.

### l-DNA aptamer western blot

The Cy5-l-13t aptamer was heated to 85 °C for 5 min in physiological buffer, slowly cooled to 25 °C over 10 min for annealing. Native human thrombin was separated by 15% SDS–PAGE and transferred to a nitrocellulose membrane in 1× transfer buffer (25 mM Tris, 192 mM glycine, 20% (v/v) methanol, pH 8.3). The membrane was incubated in 1× blocking buffer (137 mM NaCl, 2.7 mM KCl, 10 mM Na_2_HPO_4_, 1.8 mM KH_2_PO_4_, 25 mg ml^−1^ bovine serum albumin, 0.05% (v/v) Tween 20, pH 7.4) at room temperature for 1 h and incubated with 500 nM Cy5-l-13t aptamer in selection buffer at room temperature for 1 h. Next, the membrane was washed five times (5 min per wash) with 5 ml of selection buffer and scanned by the Amersham Typhoon Biomolecular Imager under the Cy5 mode. Traditional western blot was performed with mouse monoclonal primary antibody targeting native human thrombin (1:500 dilution) and Alexa Fluor 647-labeled goat anti-mouse IgG polyclonal secondary antibody (1:1,000 dilution) according to the manufacturer’s instructions (Abcam).

### Reporting summary

Further information on research design is available in the Nature Research Reporting Summary linked to this article.

## Online content

Any methods, additional references, Nature Research reporting summaries, source data, extended data, supplementary information, acknowledgements, peer review information; details of author contributions and competing interests; and statements of data and code availability are available at 10.1038/s41587-022-01337-8.

## Supplementary information

Supplementary Information Supplementary Figs. 1–19 and Tables 1–6.

Reporting summary

## Data Availability

The data that support the findings of this study are available within the paper and the Supplementary Information.

## Extended data

are available for this paper at 10.1038/s41587-022-01337-8.
