# Supplementary material for: Directed evolution and selection of biostable l-DNA aptamers with a mirror-image DNA polymerase
Source: Nat Biotechnol. 2022 Jun 6;40(11):1601–9. doi: 10.1038/s41587-022-01337-8 (PMC9646512; doi:10.1038/s41587-022-01337-8)
Supplement: Supplementary file 1 — Supplementary Figs. 1–19 and Tables 1–6. [file 41587_2022_1337_MOESM1_ESM.pdf]

nature portfolio

https://doi.org/10.1038/s41587-022-01337-8

Supplementary information

Directed evolution and selection of biostable L-DNA aptamers with a mirror-image DNA polymerase

In the format provided by the authors and unedited

Supplementary Information

Directed evolution and selection of biostable L-DNA aptamers with a mirror-image DNA polymerase

Ji Chen[1], Mengyin Chen[1], Ting F. Zhu[1,2,3,*]

[1]School of Life Sciences, Tsinghua-Peking Center for Life Sciences, Beijing Frontier Research Center for Biological Structure, Beijing Advanced Innovation Center for Structural Biology, Tsinghua University, Beijing, China

[2]School of Life Sciences, Westlake University, Hangzhou, Zhejiang, China

[3]Westlake Laboratory of Life Sciences and Biomedicine, Hangzhou, Zhejiang, China

*To whom correspondence should be addressed. ORCID: 0000-0003-0897-0303, E-mail: tzhu@westlake.edu.cn

Contents **Page**

Supplementary Figs. 1-19 2-21
Supplementary Tables 1-6 22-28

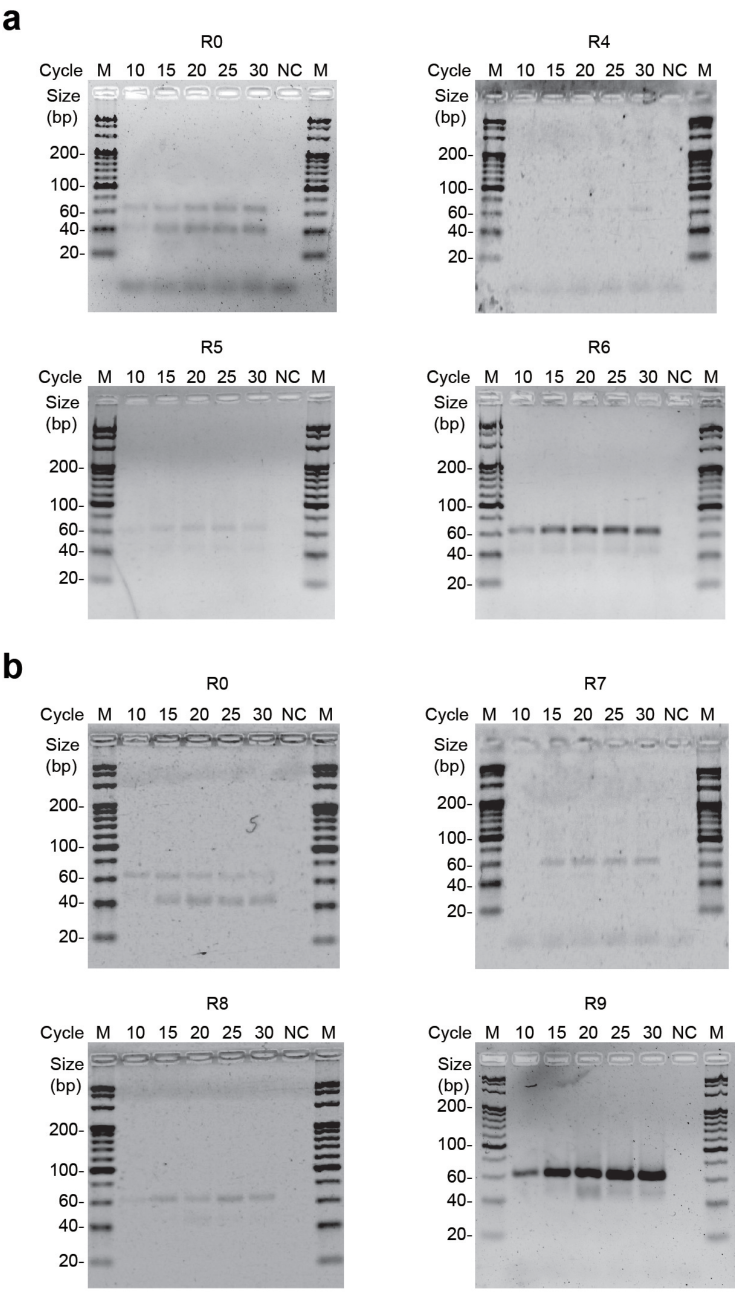

Supplementary Fig. 1 | Amplifying D- or L-DNA library and pools by natural or mirror-image PCR. a, The unselected R0 D-DNA library and R4-R6 D-DNA pools were amplified by natural PCR using L-Dpo4-5m with D-dNTPs and D-DNA primers, analyzed by 3% sieving agarose gel electrophoresis, stained by ExRed, and scanned by the ChemiDoc XRS+ system, with cycle numbers indicated above the lanes. NC, negative control without L-Dpo4-5m. The experiments were performed once. **b**, The unselected R0 L-DNA library and R7-R9 L-DNA pools were amplified by mirror-image PCR using D-Dpo4-5m with L-dNTPs and L-DNA primers, analyzed by 3% sieving agarose gel electrophoresis, stained by ExRed, and scanned by the ChemiDoc XRS+ system, with cycle numbers indicated above the lanes. NC, negative control without D-Dpo4-5m. The experiments were performed once. M, DNA marker.

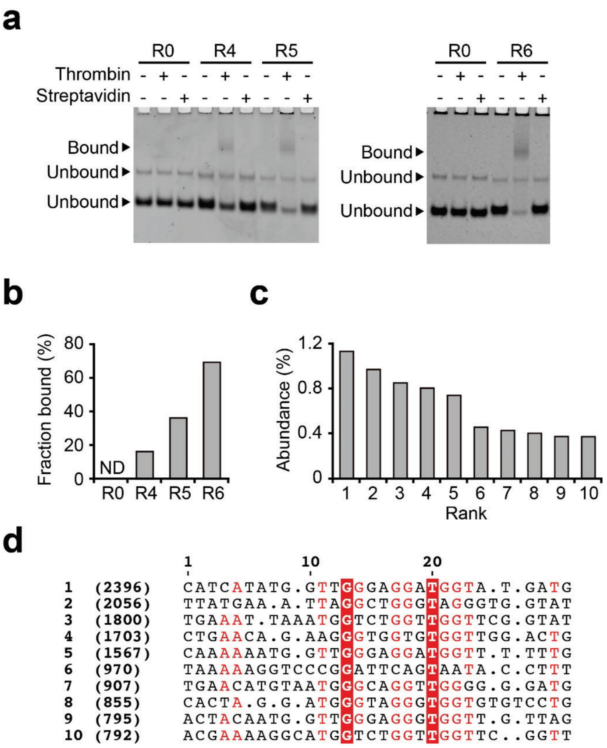

Supplementary Fig. 2 | Selecting D-DNA aptamers targeting native human thrombin. a, Monitoring the progress of selection by EMSA with 200 nM corresponding D-DNA library and pools binding with 1 µM native human thrombin or 1 µM streptavidin, analyzed by 8% native PAGE, stained by SYBR Green II, and scanned by the Amersham Typhoon Biomolecular Imager under the Cy2 mode. The experiments were performed once. **b**, Gel quantitation results of (**a**), with the fraction bound determined by the ImageJ software using the band intensity of the bound D-DNA pool relative to the total lane fluorescence intensity. **c**, Percent abundance of the top ten most abundant sequences revealed by high-throughput sequencing of the R6 D-DNA pool. The experiment was performed once. **d**, Sequence alignment of the top ten most abundant sequences in the R6 D-DNA pool, with the conserved nucleotides highlighted in red and number of reads indicated in parentheses (among 210642 total reads).

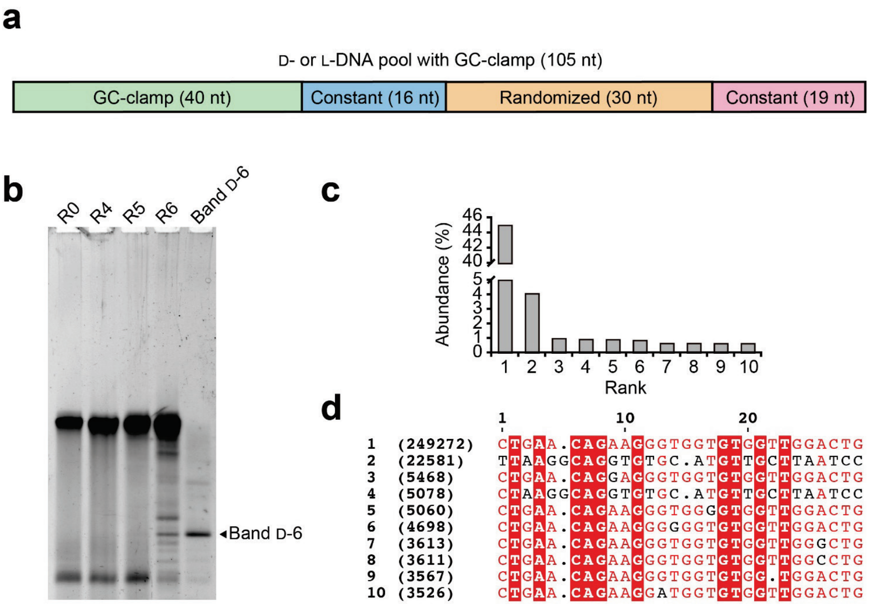

Supplementary Fig. 3 | Isolating single D-DNA aptamer sequences by DGGE. a, Design of the D- or L-DNA pool for DGGE analysis in this work. **b**, DGGE analysis of the corresponding D-DNA library and pools, as well as the isolated band D-6, re-amplified by natural PCR using L-Dpo4-5m with D-dNTPs and D-DNA primers, analyzed by 7.5 % denaturing PAGE in 2.1-4.2 M urea and 12-24% formamide, stained by SYBR Green II, and scanned by the Amersham Typhoon Biomolecular Imager under the Cy2 mode. The experiment was performed twice with similar results. **c**, Percent abundance of the top ten most abundant sequences revealed by high-throughput sequencing of band D-6. The experiment was performed once. **d**, Sequence alignment of the top ten most abundant sequences in band D-6, with the conserved nucleotides highlighted in red and number of reads indicated in parentheses (among 554081 total reads). Note that the most abundant sequence in band D-6 (with 249272 reads) is the fourth most abundant sequence in the R6 D-DNA pool (with 1703 reads among 210642 total reads, as show in Supplementary Fig. 2d).

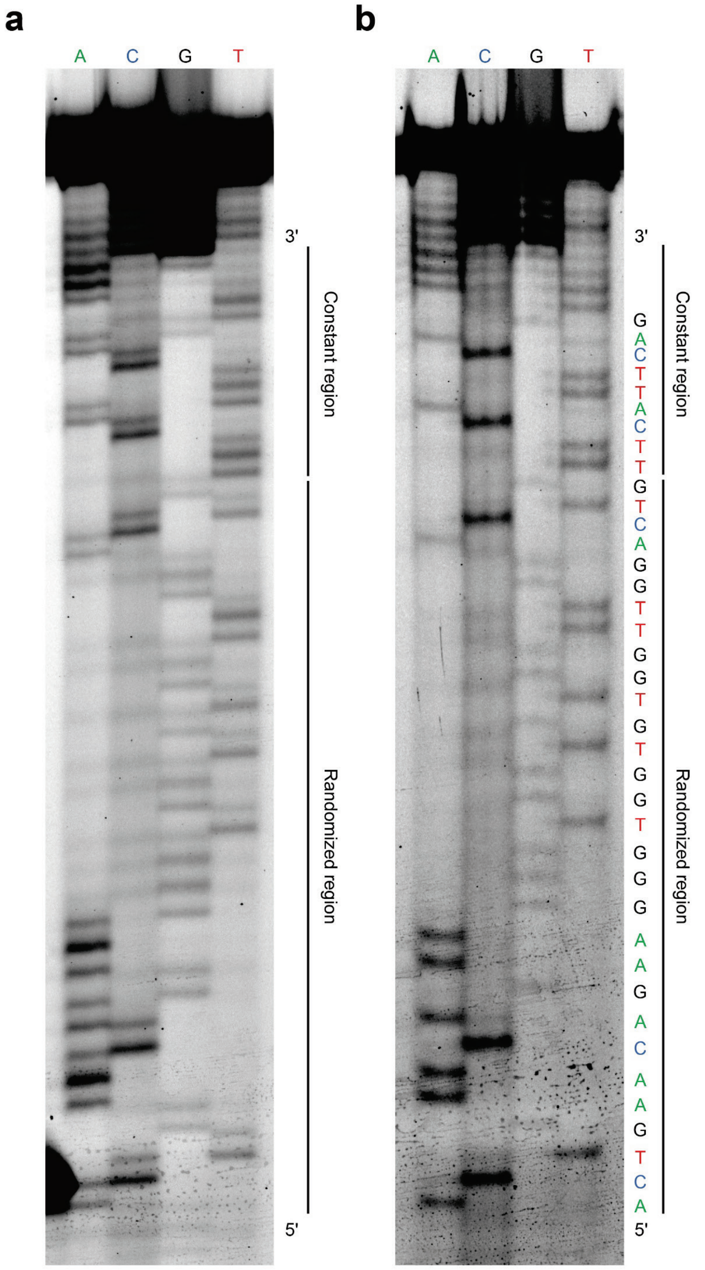

Supplementary Fig. 4 | Sequencing the DGGE-isolated D-DNA aptamer using the phosphorothioate approach. a, Band D-6 amplified by natural PCR using L-Dpo4-5m with D-dNTPαSs and 5'-FAM-labeled D-DNA forward sequencing primer, cleaved by 2-iodoethanol, analyzed by 10% denaturing PAGE in 7 M urea, and scanned by the Amersham Typhoon Biomolecular Imager under the Cy2 mode. The experiment was performed twice with similar results. **b**, Band D-6 amplified by natural PCR using L-Dpo4-5m with D-dNTPαSs and 5'-FAM-labeled D-DNA forward sequencing primer, cleaved by 2-iodoethanol, treated by natural CIP, analyzed by 10% denaturing PAGE in 7 M urea, and scanned by the Amersham Typhoon Biomolecular Imager under the Cy2 mode. The experiment was performed twice with similar results.

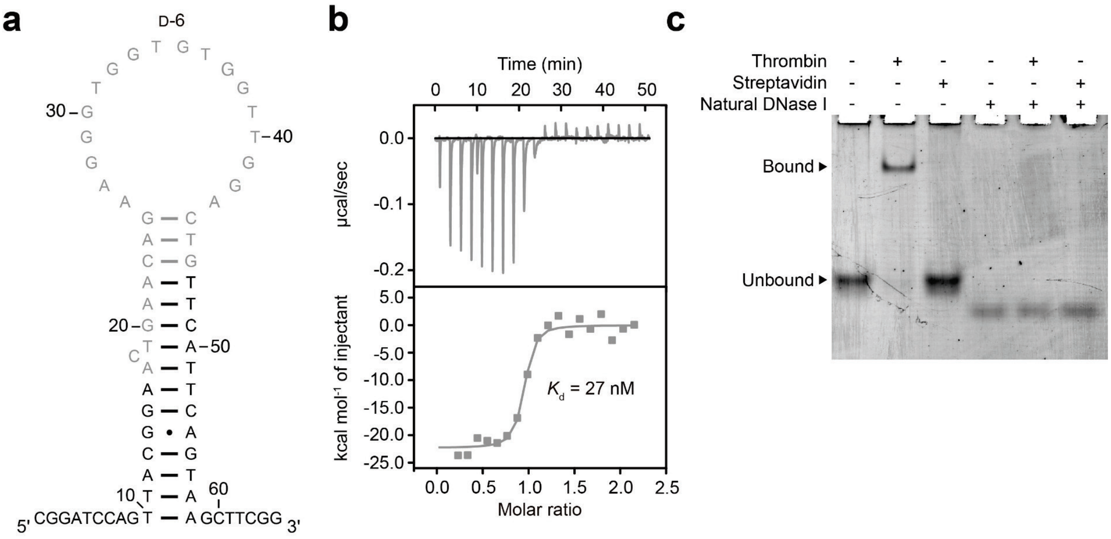

Supplementary Fig. 5 | Characterizing the selected D-DNA aptamer. a, Secondary structure of the D-6 aptamer predicted by Mfold, with nucleotides derived from the randomized region shown in gray. **b**, ITC analysis of the D-6 aptamer binding with native human thrombin, with K_d measured at 27 nM. The experiment was performed twice with similar results. **c**, EMSA of 200 nM D-6 aptamer binding with 1 μM native human thrombin or 1 μM streptavidin, without or with 50 units ml^{-1} natural DNase I, analyzed by 8% native PAGE, stained by SYBR Green II, and scanned by the Amersham Typhoon Biomolecular Imager under the Cy2 mode. The experiment was performed twice with similar results.

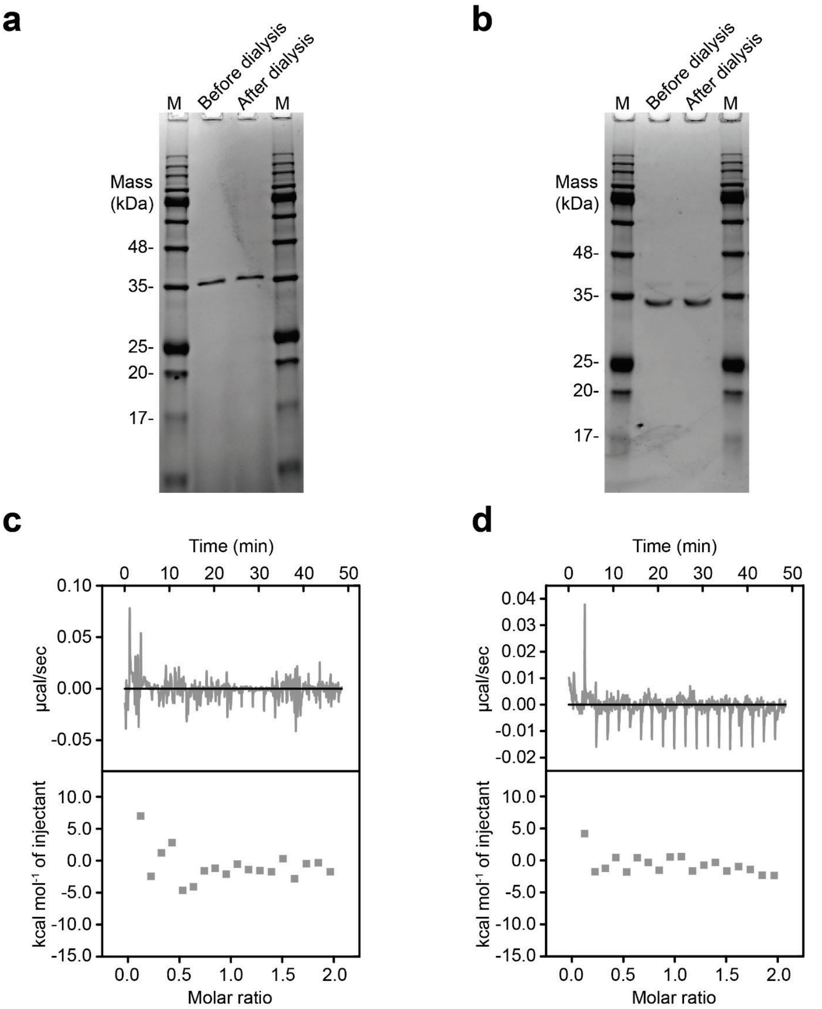

Supplementary Fig. 6 | Ruling out potential autolysis of native human and bovine thrombin. a,**b**, Native human (**a**) or bovine (**b**) thrombin before and after dialysis, separated by 15% SDS-PAGE, stained by Coomassie brilliant blue, and scanned by the ChemiDoc XRS+ system. M, prestained protein marker labeled with fluorescent dye. The experiments were performed twice with similar results. **c**,**d**, ITC analysis of physiological buffer alone added to 7 µM native human (**c**) or bovine (**d**) thrombin. The experiments were performed twice with similar results.

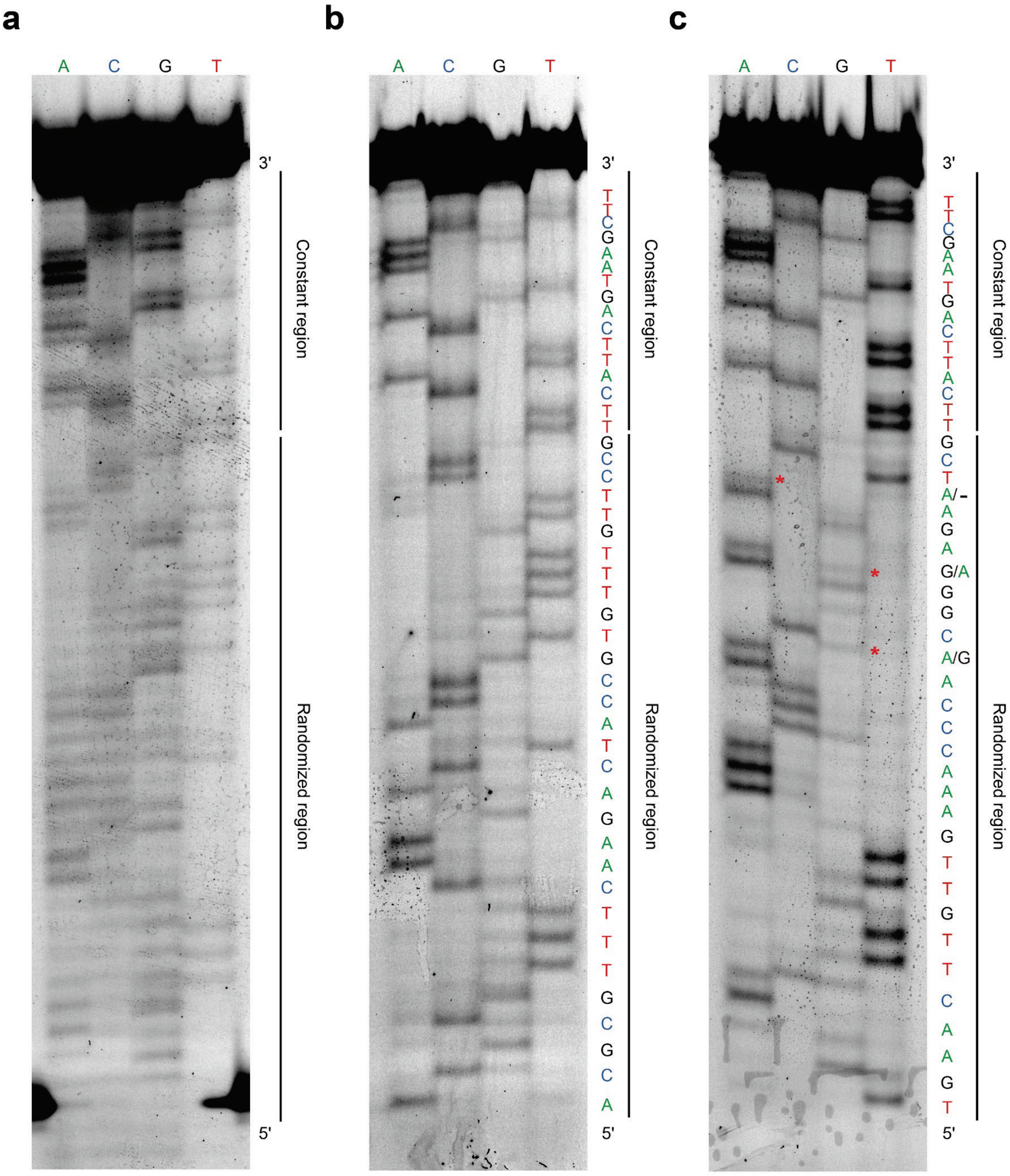

Supplementary Fig. 7 | Sequencing the DGGE-isolated L-DNA aptamers using the phosphorothioate approach. a, Band L-9-1 amplified by mirror-image PCR using D-Dpo4-5m with L-dNTPαSs and 5'-FAM-labeled L-DNA forward sequencing primer, cleaved by 2-iodoethanol, analyzed by 10% denaturing PAGE, and scanned by the Amersham Typhoon Biomolecular Imager under the Cy2 mode. The experiment was performed twice with similar results. **b**,**c**, Bands L-9-1 (**b**) and L-9-2 (**c**) amplified by mirror-image PCR using D-Dpo4-5m with L-dNTPαSs and 5'-FAM-labeled L-DNA forward sequencing primer, cleaved by 2-iodoethanol, treated by natural CIP, analyzed by 10% denaturing PAGE, and scanned by the Amersham Typhoon Biomolecular Imager under the Cy2 mode, with the corresponding chromatograms shown in Fig. 1e and f, respectively. The three ambiguous nucleotide positions in the randomized region of the sequenced L-9-2 aptamer are labeled with asterisks with the most probable alternative nucleotides (A and G) or deletion (-) indicated. The experiments were performed twice with similar results.

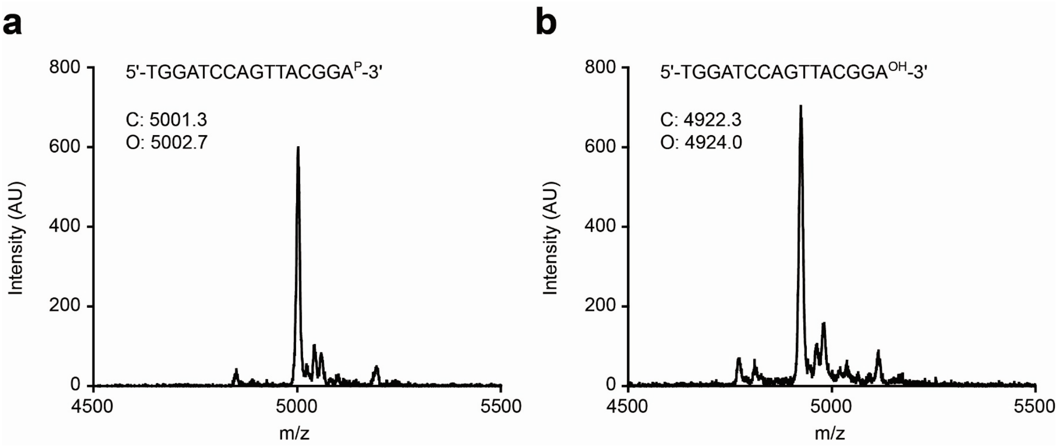

Supplementary Fig. 8 | Dephosphorylation of L-DNA by natural CIP. a,**b**, MALDI-TOF MS analysis of 3'-monophosphate-labeled L-DNA oligo before (**a**) and after (**b**) natural CIP treatment. AU, arbitrary unit. C, O, calculated and observed m/z values, respectively. Calculated m/z values are based on the molecular mass of L-DNA oligos with 5'-hydroxyl termini. The experiment was performed once.

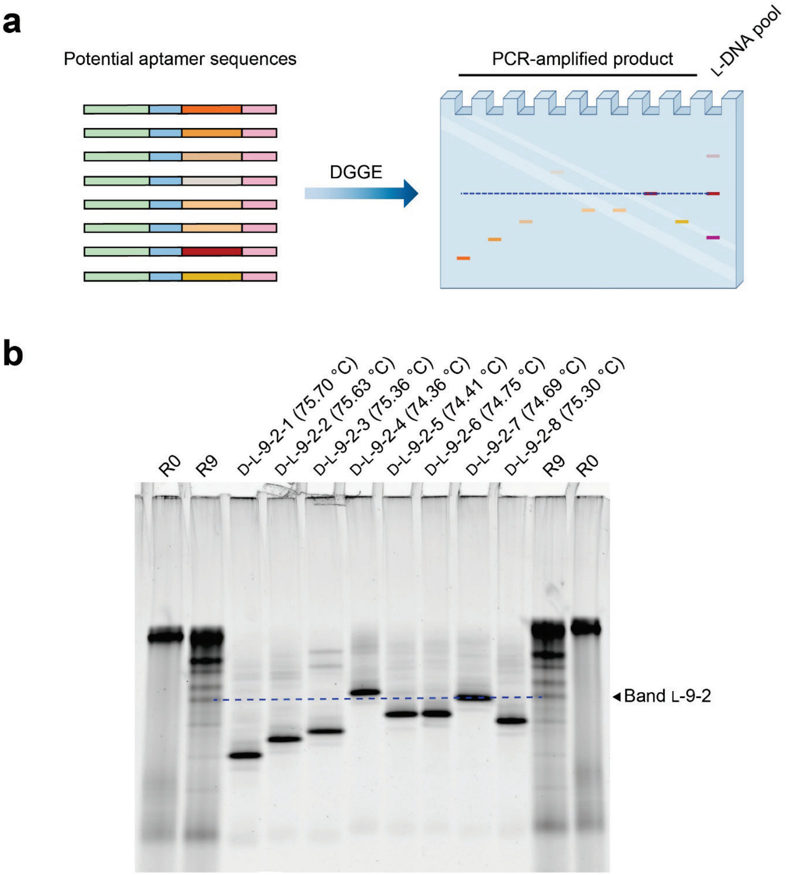

Supplementary Fig. 9 | Ruling out incorrect sequences from the band L-9-2 sequencing results by DGGE. a, Schematic overview of ruling out incorrect sequences by DGGE, since the correct sequence(s) should comigrate with band L-9-2 for the identical T_m. **b**, Natural versions of the eight most probable L-DNA aptamer sequences (Supplementary Table 1) in band L-9-2 (D-L-9-2-1 to D-L-9-2-8, with calculated T_m indicated in parentheses) amplified by natural PCR using the FastPfu Fly DNA polymerase with D-dNTPs and D-DNA primers, along with the unselected R0 L-DNA library and R9 L-DNA pool, analyzed by 10% denaturing PAGE in 2.1-4.2 M urea and 12-24% formamide, stained by SYBR Green II, and scanned by the Amersham Typhoon Biomolecular Imager under the Cy2 mode, with comigration of D-L-9-2-7 and band L-9-2 indicated by a straight dashed blue line. The experiment was performed twice with similar results.

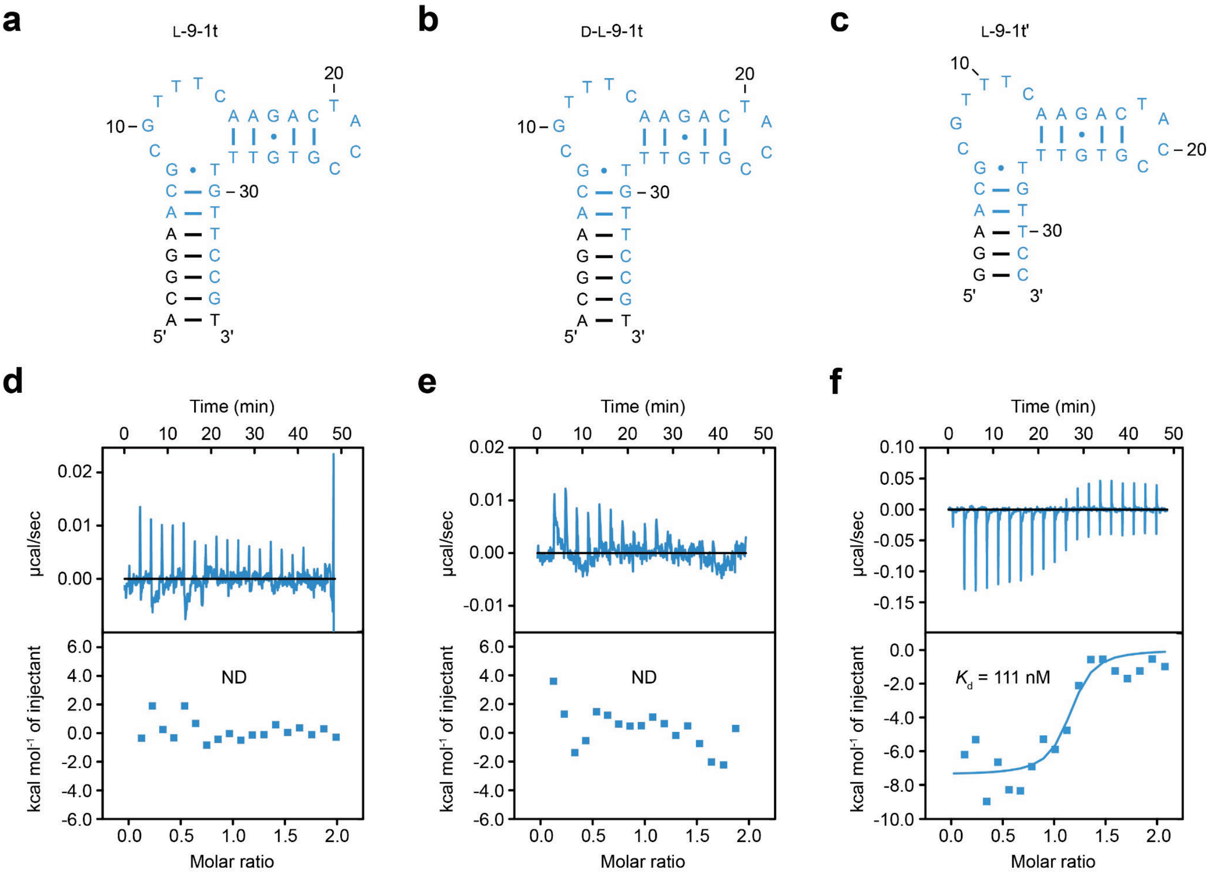

Supplementary Fig. 10 | Characterizing the truncated L-9-1t aptamer. a-c, Secondary structures of the L-9-1t aptamer (**a**, truncated version), the natural version of the L-9-1t aptamer (**b**, D-L-9-1t), and the L-9-1t' aptamer (**c**, further truncated version) predicted by Mfold, with nucleotides derived from the randomized region shown in cyan. **d**, ITC analysis of the L-9-1t aptamer binding with streptavidin. ND, (binding) not detected. The experiment was performed twice with similar results. **e**, ITC analysis of D-L-9-1t binding with native human thrombin. ND, (binding) not detected. The experiment was performed twice with similar results. **f**, ITC analysis of the L-9-1t' aptamer binding with native human thrombin, with K_d measured at 111 nM. The experiment was performed twice with similar results.

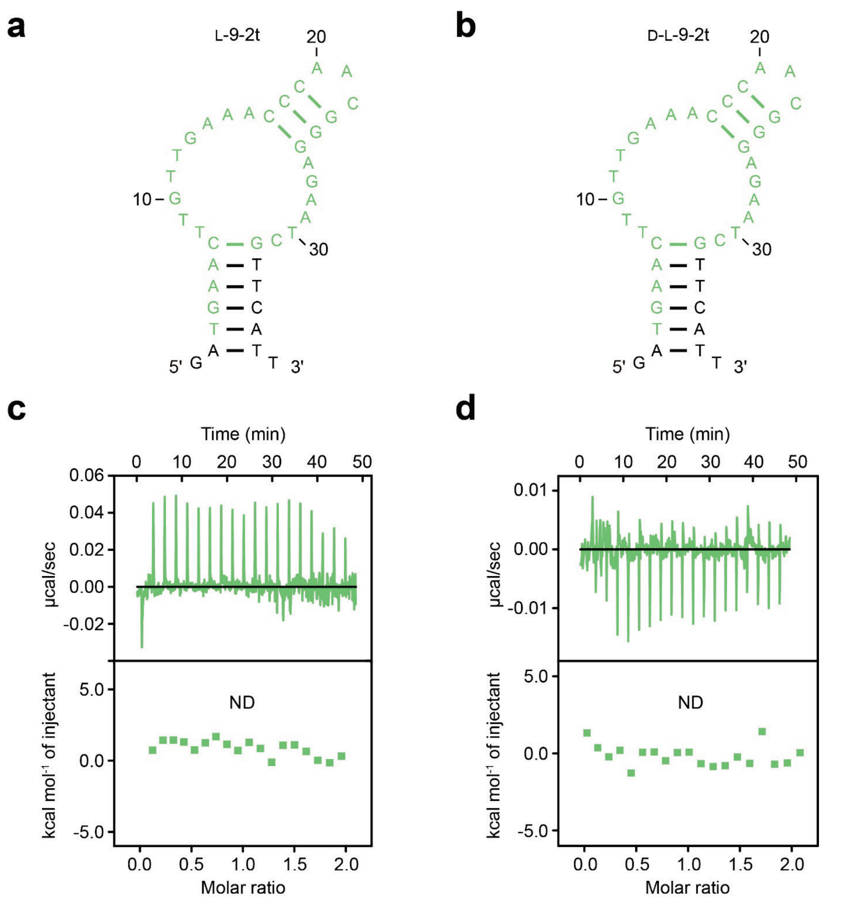

Supplementary Fig. 11 | Characterizing the truncated L-9-2t aptamer. a,**b**, Secondary structures of the L-9-2t aptamer (**a**, truncated version) and the natural version of the L-9-2t aptamer (**b**, D-L-9-2t) predicted by Mfold, with nucleotides derived from the randomized region shown in light green. **c**, ITC analysis of the L-9-2t aptamer binding with streptavidin. ND, (binding) not detected. The experiment was performed twice with similar results. **d**, ITC analysis of D-L-9-2t binding with native human thrombin. ND, (binding) not detected. The experiment was performed twice with similar results.

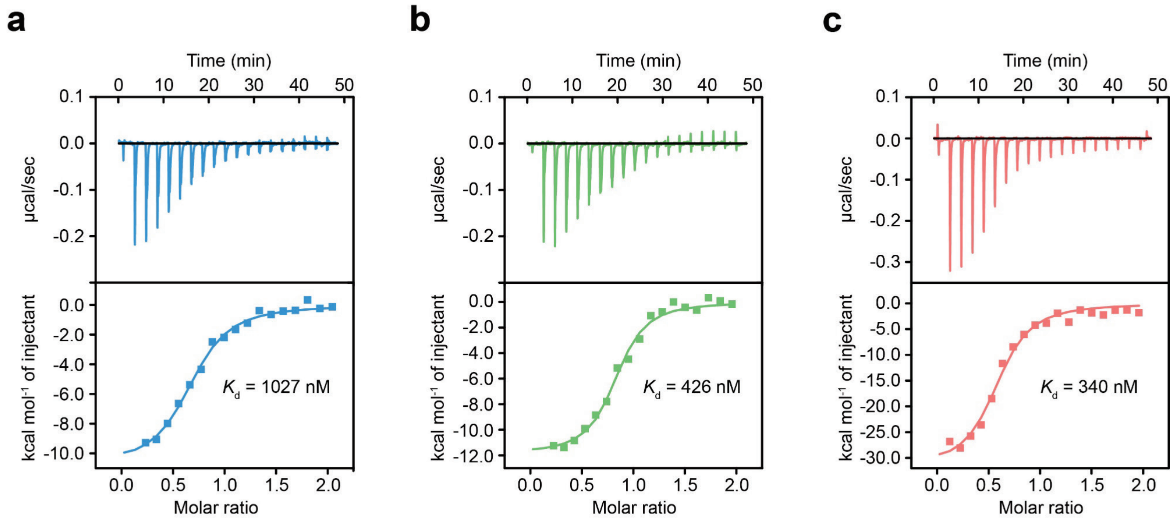

Supplementary Fig. 12 | Characterizing the binding of L-DNA aptamers with native bovine thrombin. a-c, ITC analysis of the L-9-1t (**a**), L-9-2t (**b**), and L-13t (**c**) aptamers binding with native bovine thrombin, with K_d measured at 1027, 426, and 340 nM, respectively. The experiments were performed once.

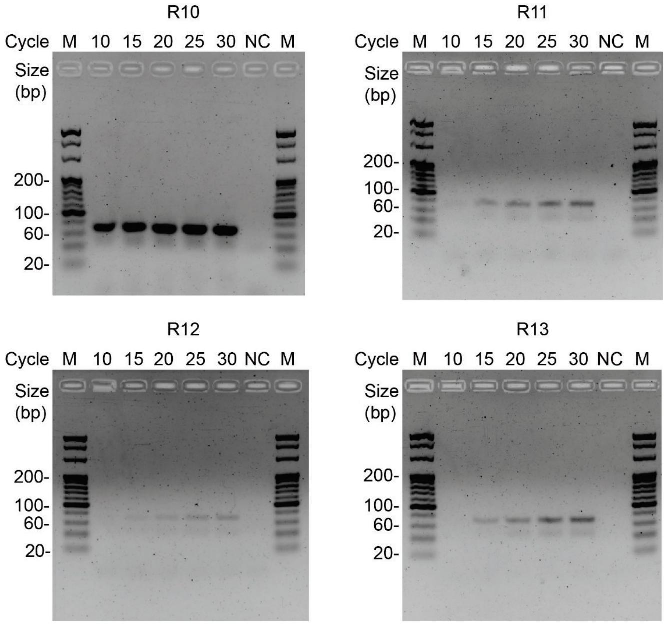

Supplementary Fig. 13 | Amplifying partially randomized L-DNA library and pools by mirror-image PCR. The partially randomized R10 L-DNA library and R11-R13 L-DNA pools were amplified by mirror-image PCR using D-Dpo4-5m with L-dNTPs and L-DNA primers, analyzed by 3% sieving agarose gel electrophoresis, stained by ExRed, and scanned by the ChemiDoc XRS+ system, with cycle numbers indicated above the lanes. NC, negative control without D-Dpo4-5m. M, DNA marker. The experiments were performed once.

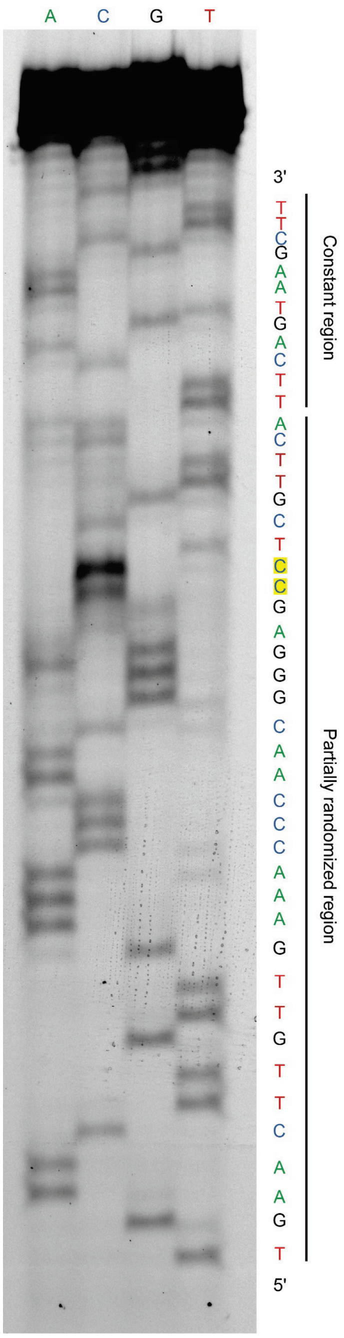

Supplementary Fig. 14 | Sequencing the DGGE-isolated L-13 aptamer using the phosphorothioate approach. Band L-13 amplified by mirror-image PCR using D-Dpo4-5m with L-dNTPαSs and 5'-FAM-labeled L-DNA forward sequencing primer, cleaved by 2-iodoethanol, treated by natural CIP, analyzed by 10% denaturing PAGE, and scanned by the Amersham Typhoon Biomolecular Imager under the Cy2 mode, with the two mutations highlighted in yellow and the corresponding chromatogram shown in Fig. 3d. The experiment was performed twice with similar results.

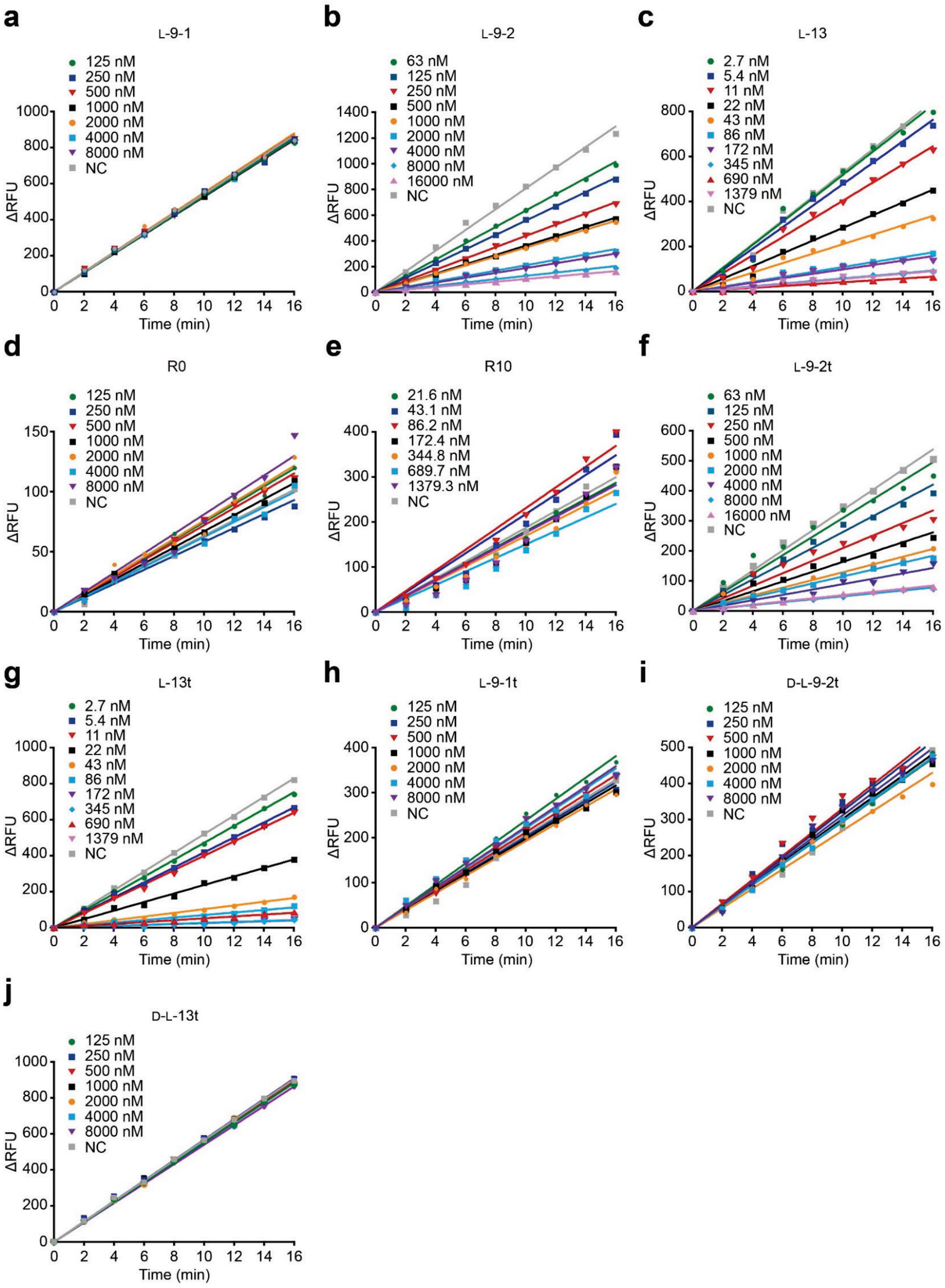

Supplementary Fig. 15 | Testing the inhibition of native human thrombin enzymatic activity. a-i, Measured relative fluorescence of the L-9-1 (**a**), L-9-2 (**b**), and L-13 (**c**) aptamers, the unselected R0 (**d**), and the partially randomized R10 (**e**) L-DNA libraries, the L-9-2t (**f**), L-13t (**g**), and L-9-1t (**h**) aptamers, as well as the natural versions of the L-9-2t and L-13t aptamers (D-L-9-2t (**i**) and D-L-13t (**j**), respectively), incubated with 10 nM native human thrombin and 100 μM fluorogenic substrate, benzoyl-Phe-Val-Arg-AMC, in physiological buffer for up to 16 min, with excitation wavelength at 350 nm and emission wavelength at 450 nm, and measurements taken every 2 min. RFU, relative fluorescence unit. ΔRFU, change of relative fluorescence unit with RFU measured at 0 min set to 0. NC, negative control with 100 μM benzoyl-Phe-Val-Arg-AMC in physiological buffer alone. The experiments were performed twice with similar results.

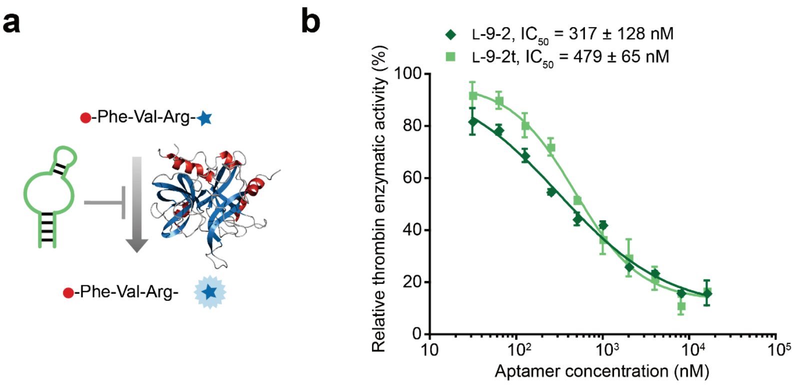

Supplementary Fig. 16 | L-DNA aptamer thrombin inhibitors. a, Schematic overview of inhibiting native human thrombin enzymatic activity with the selected L-DNA aptamers, native human thrombin, and the fluorogenic substrate, benzoyl-Phe-Val-Arg-AMC. **b**, Relative thrombin enzymatic activities of 10 nM native human thrombin and 100 μM benzoyl-Phe-Val-Arg-AMC, incubated with the L-9-2 and L-9-2t aptamers in physiological buffer, with half-maximum inhibitory concentrations (IC_{50}) measured at 317 ± 128 nM and 479 ± 65 nM, respectively. Data are presented as mean ± s.d. (n = 3, independent measurements) with aptamer concentration shown in logarithmic scale.

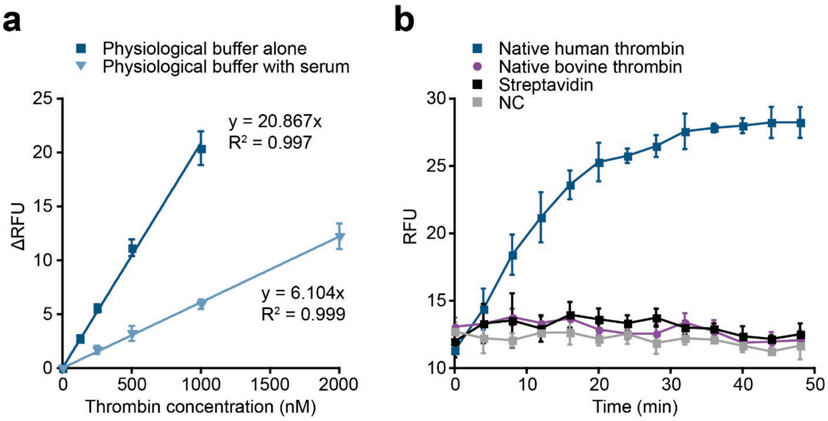

Supplementary Fig. 17 | L-DNA aptamer sensor. a, Standard curves for measuring native human thrombin concentrations, with the L-DNA aptamer sensor incubated with various concentrations of native human thrombin in physiological buffer or physiological buffer with 10% human serum. Data are presented as mean ± s.d. (n = 3, independent measurements), with the slope and coefficient of determination (R^2) calculated by the KaleidaGraph software using linear regression. **b**, Measured relative fluorescence of the L-DNA aptamer sensor incubated with 1 μM native human thrombin, 1 μM native bovine thrombin, or 1 μM streptavidin in physiological buffer, with excitation wavelength at 494 nm and emission wavelength at 518 nm, and measurements taken every 4 min. NC, negative control with the L-DNA aptamer sensor in physiological buffer alone. RFU, relative fluorescence unit. ΔRFU, change of relative fluorescence unit with RFU measured with the L-DNA aptamer sensor in physiological buffer alone set to 0. Data are presented as mean ± s.d. (n = 3, independent measurements).

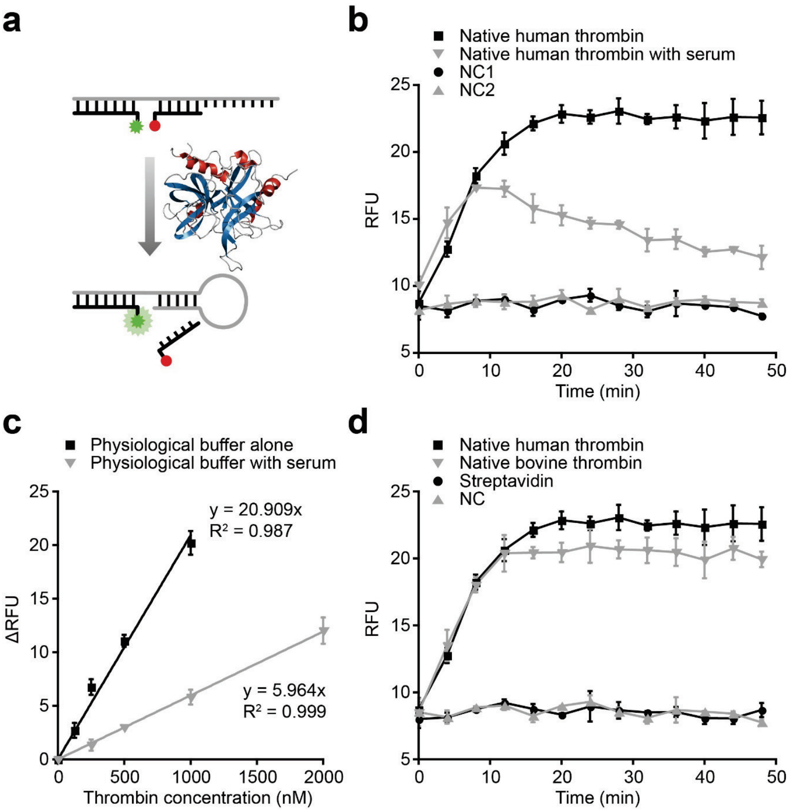

Supplementary Fig. 18 | D-DNA aptamer sensor. a, Schematic overview of detecting native human thrombin with the D-DNA aptamer sensor based on the D-6 aptamer. **b**, Measured relative fluorescence of the D-DNA aptamer sensor incubated with 1 μM native human thrombin in physiological buffer alone, or physiological buffer with 10% human serum for up to 48 min, with excitation wavelength at 494 nm and emission wavelength at 518 nm, and measurements taken every 4 min. NC1, negative control with the D-DNA aptamer sensor in physiological buffer alone. NC2, negative control with the D-DNA aptamer sensor in physiological buffer with 10% human serum. Data are presented as mean ± s.d. (n = 3, independent measurements). **c**, Standard curves for measuring native human thrombin concentrations, with the D-DNA aptamer sensor incubated with various concentrations of native human thrombin in physiological buffer or physiological buffer with 10% human serum. Data are presented as mean ± s.d. (n = 3, independent measurements), with the slope and coefficient of determination (R^2) calculated by the KaleidaGraph software using linear regression. **d**, Measured relative fluorescence of the D-DNA aptamer sensor incubated with 1 μM native human thrombin, 1 μM native bovine thrombin, or 1 μM streptavidin in physiological buffer, with excitation wavelength at 494 nm and emission wavelength at 518 nm, and measurements taken every 4 min. NC, negative control with the D-DNA aptamer sensor in physiological buffer alone. RFU, relative fluorescence unit. ΔRFU, change of relative fluorescence unit with RFU measured with the D-DNA aptamer sensor in physiological buffer alone set to 0. Data are presented as mean ± s.d. (n = 3, independent measurements).

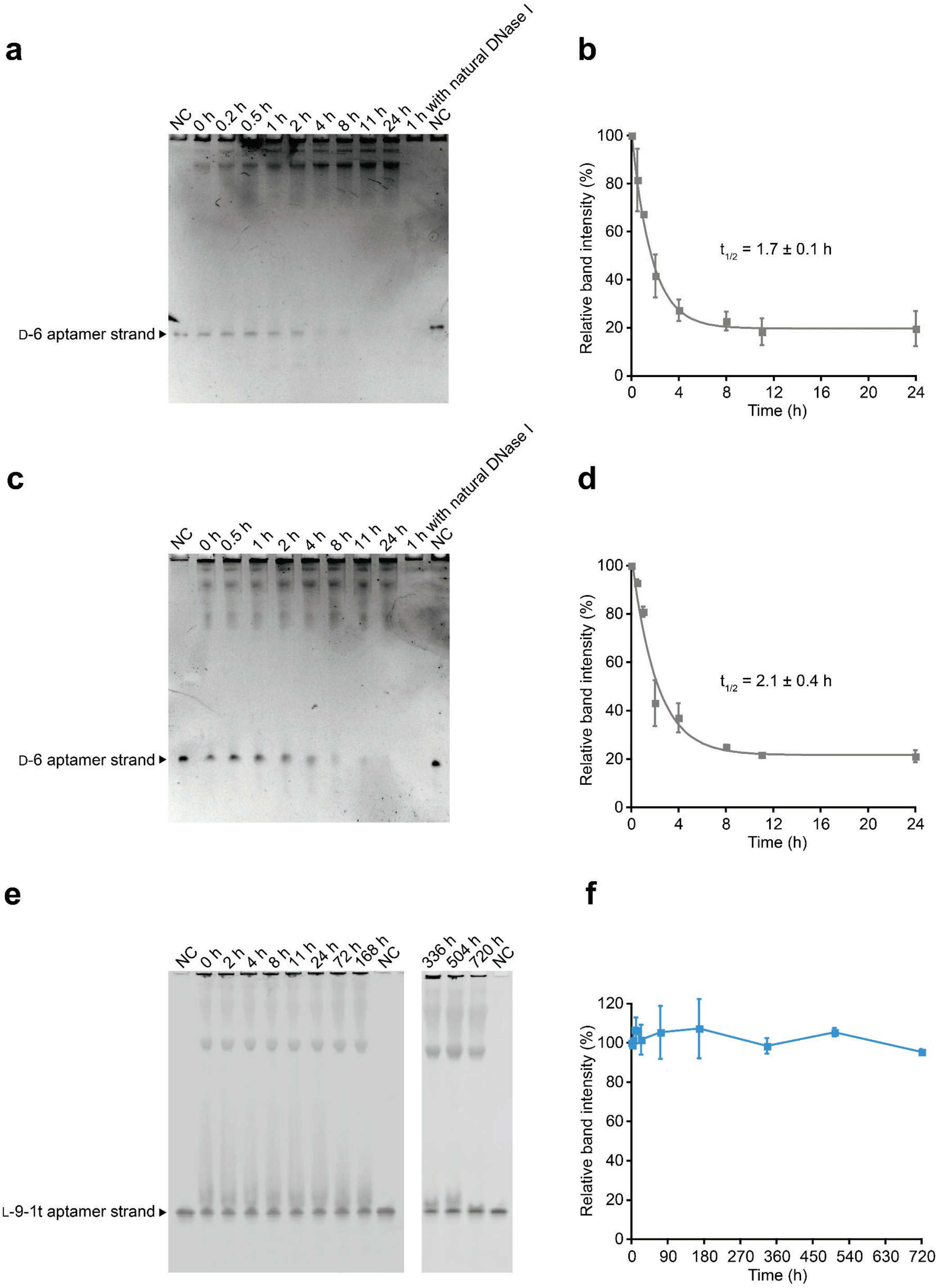

Supplementary Fig. 19 | Biostability of the D- and L-DNA aptamer sensors. a, D-DNA aptamer sensor incubated in physiological buffer with 10% human serum (or 50 units ml^{-1} natural DNase I), sampled at selected time points, analyzed by 10% denaturing PAGE in 7 M urea, stained by SYBR Green II, and scanned by the ChemiDoc XRS+ system. The experiment was performed three times with similar results. **b**, Gel quantitation results of (**a**), with the full-length band intensity of the D-DNA aptamer strand at selected time points normalized by that of 0 h, and half-life ($t_{1/2}$) measured at 1.7 ± 0.1 h. Data are presented as mean ± s.d. (n = 3, independent measurements). **c**, D-DNA aptamer sensor incubated in

physiological buffer with 83% human serum (or 50 units ml^{-1} natural DNase I), sampled at selected time points, analyzed by 10% denaturing PAGE in 7 M urea, stained by SYBR Green II, and scanned by the ChemiDoc XRS+ system. The experiment was performed three times with similar results. **d**, Gel quantitation results of (**c**), with the full-length band intensity of the D-DNA aptamer strand at selected time points normalized by that of 0 h, and $t_{1/2}$ measured at 2.1 ± 0.4 h. Data are presented as mean ± s.d. (n = 3, independent measurements). **e**, L-DNA aptamer sensor incubated in physiological buffer with 83% human serum, sampled at selected time points, analyzed by 10% denaturing PAGE in 7 M urea, stained by SYBR Green II, and scanned by the ChemiDoc XRS+ system. The experiment was performed three times with similar results. **f**, Gel quantitation results of (**e**), with the full-length band intensity of the L-DNA aptamer strand at selected time points normalized by that of 0 h, and degradation not detected. NC, negative control with the D- or L-DNA aptamer sensor incubated in physiological buffer alone and sampled at 1 h. Data are presented as mean ± s.d. (n = 3, independent measurements).

Supplementary Table 1 | D-DNA oligos for DGGE analysis with calculated melting temperature (T_m)

Oligo	Sequence	T_m (°C)
D-L-9-2-1	5'-CGGATCCAGTTACGGATGAACTTGTTGAAACCCAGCGGGAG ATCGTTCATTCAGTAAGCTTCGG-3'	75.70
D-L-9-2-2	5'-CGGATCCAGTTACGGATGAACTTGTTGAAACCCAGCGGGAG AATCGTTCATTCAGTAAGCTTCGG-3'	75.63
D-L-9-2-3	5'-CGGATCCAGTTACGGATGAACTTGTTGAAACCCAGCGGAAG ATCGTTCATTCAGTAAGCTTCGG-3'	75.36
D-L-9-2-4	5'-CGGATCCAGTTACGGATGAACTTGTTGAAACCCAACGGAAG AATCGTTCATTCAGTAAGCTTCGG-3'	74.36
D-L-9-2-5	5'-CGGATCCAGTTACGGATGAACTTGTTGAAACCCAACGGAAG ATCGTTCATTCAGTAAGCTTCGG-3'	74.41
D-L-9-2-6	5'-CGGATCCAGTTACGGATGAACTTGTTGAAACCCAACGGGAG ATCGTTCATTCAGTAAGCTTCGG-3'	74.75
D-L-9-2-7	5'-CGGATCCAGTTACGGATGAACTTGTTGAAACCCAACGGGAG AATCGTTCATTCAGTAAGCTTCGG-3'	74.69
D-L-9-2-8	5'-CGGATCCAGTTACGGATGAACTTGTTGAAACCCAGCGGAAG AATCGTTCATTCAGTAAGCTTCGG-3'	75.30

Supplementary Table 2 | Native human thrombin concentrations measured by the D- and L-DNA aptamer sensors

Data are presented as mean ± s.d. (n = 3, independent measurements).

	Measured thrombin concentration (nM)			
Sensor	D-DNA aptamer sensor		L-DNA aptamer sensor	
Incubation time	1 h	4 h	1 h	4 h
300 nM thrombin alone	334 ± 59	424 ± 37	299 ± 13	290 ± 19
300 nM thrombin with 10% human serum	416 ± 62	784 ± 91	457 ± 72	375 ± 54
300 nM thrombin with 50 units ml^{-1} natural DNase I	1219 ± 57	984 ± 52	334 ± 58	251 ± 34

Supplementary Table 3 | Conditions for D-DNA aptamer selection

Round	Amount of D-DNA pool (pmol)	Thrombin-coupled bead volume (µl)	Incubation volume (µl)	Wash condition	Natural PCR volume (µl)	Number of natural PCR cycles
1	600	100	400	10 s × 3	1500	20
2	200	50	280	10 s × 4	1500	25
3	200	50	250	5 min × 4	1500	30
4	100	20	200	5 min × 5	1000	30
5	100	20	200	10 min × 5	1000	20
6	50	10	130	10 min × 6	500	15

Supplementary Table 4 | Conditions for L-DNA aptamer selection

Round	Amount of L-DNA pool (pmol)	Thrombin-coupled bead volume (μl)	Incubation volume (μl)	Wash condition	Mirror-image PCR volume (μl)	Number of mirror-image PCR cycles
1	600	100	400	10 s × 3	2500	20
2	200	50	280	10 s × 4	1500	20
3	200	50	250	5 min × 4	1500	30
4	100	20	200	5 min × 5	1000	30
5	100	20	200	5 min × 6	1000	30
6	50	10	130	7 min × 6	500	25
7	50	5	190	10 min × 6	500	15
8	30	5	170	10 min × 8	500	15
9	30	3	200	10 min × 8	500	10

Supplementary Table 5 | Conditions for the secondary selection of L-DNA aptamers

Round	Amount of L-DNA pool (pmol)	Thrombin-coupled bead volume (μl)	Incubation volume (μl)	Wash condition	Mirror-image PCR volume (μl)	Number of mirror-image PCR cycles
11	200	50	300	5 min × 3	1000	20
12	100	10	400	7 min × 6	800	20
13	50	5	400	10 min × 6	500	15

Supplementary Table 6 | DNA oligo sequences

N's denote nucleotides with full randomization
Lower-case letters denote nucleotides with partial randomization at a frequency of 10%

Oligo	Sequence
D- or L-DNA library	5'-CGGATCCAGTTACGGANNNNNNNNNNNNNNNNNNNNNNNNNNNNNNNTTCATTCAGTAAGCTTCGG-3'
D- or L-DNA forward primer	5'-CGGATCCAGTTACGGA-3'
D- or L-DNA reverse primer	5'-CCGAAGCTTACTGAATGAA-3'
D- or L-DNA reverse primer with Sp18	5'-AAAAAAAAAAAAAAAAAAAA-Sp18-CCGAAGCTTACTGAATGAA-3'
D- or L-DNA forward primer with GC-clamp	5'-CGCCCGCCGCGCCCCGCGCCCGTCCCGCCGCCCCCGCCCGCGGATCCAGTTACGGA-3'
D- or L-DNA forward sequencing primer	5'-FAM-CGCCCGCCGCGCCCCGCGCCCGTCCCGCCGCCCCCGCCCGCGGATCCAGTTACGGA -3'
3'-monophosphate labeled L-DNA	5'-TGGATCCAGTTACGGA^{P}-3'
D-6 aptamer	5'-CGGATCCAGTTACGGAACTGAACAGAAGGGTGGTGTGGTTGGACTGTTCATTCAGTAAGCTTCGG-3'
L-9-1 aptamer	5'-CGGATCCAGTTACGGAACGCGTTTCAAGACTACCGTGTTTGTTCCGTTCATTCAGTAAGCTTCGG-3'
L-9-2 aptamer	5'-CGGATCCAGTTACGGATGAACTTGTTGAAACCCAACGGGAGAATCGTTCATTCAGTAAGCTTCGG-3'
L-9-1t aptamer	5'-ACGGAACGCGTTTCAAGACTACCGTGTTTGTTCCGT-3'
L-9-2t aptamer	5'-GATGAACTTGTTGAAACCCAACGGGAGAATCGTTCATT-3'
D-L-9-1t	5'-ACGGAACGCGTTTCAAGACTACCGTGTTTGTTCCGT-3'
D-L-9-2t	5'-GATGAACTTGTTGAAACCCAACGGGAGAATCGTTCATT-3'
L-9-1t' aptamer	5'-GGAACGCGTTTCAAGACTACCGTGTTTGTTCC-3'
Cy5-L-9-1t aptamer	5'-Cy5-ACGGAACGCGTTTCAAGACTACCGTGTTTGTTCCGT-3'
Cy5-L-9-2t aptamer	5'-Cy5-GATGAACTTGTTGAAACCCAACGGGAGAATCGTTCATT-3'
Partially randomized L-DNA library	5'-CGGATCCAGTTACGGAtgaacttgttgaaacccaacgggagaatcgttcaTTCAGTAAGCTTCGGTGG-3'
L-DNA reverse primer for secondary selection	5'-CCACCGAAGCTTACTGAA-3'
L-DNA reverse primer with Sp18 for secondary selection	5'-AAAAAAAAAAAAAAAAAAAA-Sp18-CCACCGAAGCTTACTGAA-3'

L-13 aptamer	5'-CGGATCCAGTTACGGATGAACTTGTTGAAACCCAACGGGAGCCTCGTTCATTCAGTAAGCTTCGGTGG-3'
L-13t aptamer	5'-GATGAACTTGTTGAAACCCAACGGGAGCCTCGTTCATT-3'
D-L-13t	5'-GATGAACTTGTTGAAACCCAACGGGAGCCTCGTTCATT-3'
Cy5-L-13t aptamer	5'-Cy5-GATGAACTTGTTGAAACCCAACGGGAGCCTCGTTCATT-3'
D-DNA fluorophore strand	5'-FAM-TCCGTAACTGGATCCG-3'
L-DNA fluorophore strand	5'-FAM-ACTGGATCCGAGCTG-3'
D-DNA quencher strand	5'-ACCCTTCTGTTCA-DABCYL-3'
L-DNA quencher strand	5'-ACGCGTTCCGT-DABCYL-3'
D-6 aptamer strand	5'-CGGATCCAGTTACGGACTGAACAGAAGGGTGGTGTGGTTGGACTGTTCA-3'
L-9-1t aptamer strand	5'-CAGCTCGGATCCAGTTACGGAACGCGTTTCAAGACTACCGTGTTTGTTCCGT-3'
