# Supplementary material for: Directed evolution and selection of biostable l-DNA aptamers with a mirror-image DNA polymerase
Source: Nat Biotechnol. 2022 Jun 6;40(11):1601–9. doi: 10.1038/s41587-022-01337-8 (PMC9646512; doi:10.1038/s41587-022-01337-8)
Supplement: Supplementary file 2 — Reporting summary [file 41587_2022_1337_MOESM2_ESM.pdf]

nature research

Corresponding author(s): Ting Zhu

Last updated by author(s): Apr 12, 2022

Reporting Summary

Nature Research wishes to improve the reproducibility of the work that we publish. This form provides structure for consistency and transparency in reporting. For further information on Nature Research policies, see our Editorial Policies and the Editorial Policy Checklist.

Statistics

For all statistical analyses, confirm that the following items are present in the figure legend, table legend, main text, or Methods section.

n/a	Confirmed	
☐	☒	The exact sample size (*n*) for each experimental group/condition, given as a discrete number and unit of measurement
☐	☒	A statement on whether measurements were taken from distinct samples or whether the same sample was measured repeatedly
☐	☒	The statistical test(s) used AND whether they are one- or two-sided<br>*Only common tests should be described solely by name; describe more complex techniques in the Methods section.*
☒	☐	A description of all covariates tested
☒	☐	A description of any assumptions or corrections, such as tests of normality and adjustment for multiple comparisons
☐	☒	A full description of the statistical parameters including central tendency (e.g. means) or other basic estimates (e.g. regression coefficient) AND variation (e.g. standard deviation) or associated estimates of uncertainty (e.g. confidence intervals)
☐	☒	For null hypothesis testing, the test statistic (e.g. F, t, r) with confidence intervals, effect sizes, degrees of freedom and P value noted<br>*Give P values as exact values whenever suitable.*
☒	☐	For Bayesian analysis, information on the choice of priors and Markov chain Monte Carlo settings
☒	☐	For hierarchical and complex designs, identification of the appropriate level for tests and full reporting of outcomes
☒	☐	Estimates of effect sizes (e.g. Cohen's d, Pearson's r), indicating how they were calculated

Our web collection on statistics for biologists contains articles on many of the points above.

Software and code

Policy information about availability of computer code

Data collection	Agarose gels were scanned by the ChemiDoc XRS+ system. Polyacrylamide gels were scanned by the Amersham Typhoon Biomolecular Imager and the ChemiDoc XRS+ system. MALDI-TOF MS was performed by the Applied Biosystems 4800 Plus MALDI TOF/TOF Analyzer. ITC data were obtained by the MicroCal iTC200 microcalorimeter. Relative fluorescence was measured by the Varioskan Flash system. The selected D-DNA aptamers were sequenced on the Illumina HiSeq X Ten platform. Prothrombin time was measured by the STA R Max Coagulation Analyzer.
Data analysis	ImageJ (version 1.53), Microsoft Excel 2019 (version 16.43), KaleidaGraph (version 4.5.4), Origin 7 (version 7.0552).

For manuscripts utilizing custom algorithms or software that are central to the research but not yet described in published literature, software must be made available to editors and reviewers. We strongly encourage code deposition in a community repository (e.g. GitHub). See the Nature Research guidelines for submitting code & software for further information.

Data

Policy information about availability of data

All manuscripts must include a data availability statement. This statement should provide the following information, where applicable:

- Accession codes, unique identifiers, or web links for publicly available datasets
- A list of figures that have associated raw data
- A description of any restrictions on data availability

The data that support the findings of this study are available within the paper and the Supplementary Information.

April 2020

Field-specific reporting

Please select the one below that is the best fit for your research. If you are not sure, read the appropriate sections before making your selection.

☒ Life sciences ☐ Behavioural & social sciences ☐ Ecological, evolutionary & environmental sciences

For a reference copy of the document with all sections, see nature.com/documents/nr-reporting-summary-flat.pdf

Life sciences study design

All studies must disclose on these points even when the disclosure is negative.

Sample size	The sample sizes of experiments are described in the paper and the Supplementary Information. No statistical test was used to determine sample sizes.
Data exclusions	The raw Illumina reads for the high-throughput sequencing of the selected D-DNA aptamers were processed to remove the low-quality reads.
Replication	Most experiments were performed two or three times independently as described in the figure legends. All replication attempts were successful, with a representative result shown in some cases. The experiments in Fig. 1c, Extended Data Fig. 1b, and Supplementary Figs. 1a, 1b, 2a, 2c, 3c, 8, 12, 13 were performed once due to the amount of materials required.
Randomization	Randomization was not applicable to this study, as no animals or multiple human subjects were used in this study.
Blinding	Blinding was not applicable to this study, as no experiments were influenced by human interpretation.

Reporting for specific materials, systems and methods

We require information from authors about some types of materials, experimental systems and methods used in many studies. Here, indicate whether each material, system or method listed is relevant to your study. If you are not sure if a list item applies to your research, read the appropriate section before selecting a response.

Materials & experimental systems

n/a	Involved in the study	
☐	☒	Antibodies
☒	☐	Eukaryotic cell lines
☒	☐	Palaeontology and archaeology
☒	☐	Animals and other organisms
☐	☒	Human research participants
☒	☐	Clinical data
☒	☐	Dual use research of concern

Methods

n/a	Involved in the study	
☒	☐	ChIP-seq
☒	☐	Flow cytometry
☒	☐	MRI-based neuroimaging

Antibodies

Antibodies used	Mouse monoclonal primary antibody targeting native human thrombin (Abcam, catalog no. ab17199, lot GR3351446-8, clone 5G9), Alexa Fluor 647-labeled goat anti-mouse IgG polyclonal secondary antibody (Abcam, catalog no. ab150115, lot GR3399166-3).
Validation	Validation statements are available on the manufacturer's website.

Human research participants

Policy information about studies involving human research participants

Population characteristics	Human plasma was obtained with written informed consent from a 33-year-old healthy male volunteer.
Recruitment	The volunteer was recruited without self-selection bias, following the protocol approved by the Institution Review Board of Tsinghua University (project no. 20210173) with written informed consent obtained.
Ethics oversight	Institution Review Board of Tsinghua University (project no. 20210173).

Note that full information on the approval of the study protocol must also be provided in the manuscript.
